# Population structure, resistome, and virulome of *Staphylococcus chromogenes* strains from milk of subclinical bovine mastitis in South Africa

**DOI:** 10.3389/fcimb.2025.1654546

**Published:** 2025-08-22

**Authors:** N. G. Khasapane, S. J. Nkhebenyane, O. Thekisoe, T. Ramatla, K. E. Lekota

**Affiliations:** ^1^ Center for Applied Food Safety and Biotechnology, Department of Life Sciences, Central University of Technology, Bloemfontein, South Africa; ^2^ Unit for Environmental Sciences and Management, North-West University, Potchefstroom, South Africa

**Keywords:** intramammary infection, mastitis, whole genome sequencing, pangenomics, cattle

## Abstract

**Introduction:**

*Staphylococcus chromogenes* are commonly found in intramammary infections associated with bovine subclinical mastitis in dairy cattle, yet their genomic diversity and antimicrobial resistance dynamics remain poorly characterized, particularly in African settings.

**Methods:**

This study presents a comparative genomic analysis of 17 *S. chromogenes* isolates from South Africa, including five newly sequenced bovine mastitis strains and twelve porcine-derived genomes retrieved from GenBank. *In-silico* analysis using multilocus sequence typing (MLST), virulence genes, antibiotic resistance genes and plasmids replicon types were used to characterise these isolates.

**Results and discussion:**

Pairwise average nucleotide identity (ANI) analysis revealed that bovine isolates SC21, SC28, and SC33 are closely related and likely clonal members of the bovine-adapted ST138 lineage (ANI >99.7%), while SC12 and SC14 are more genetically distinct and show closer similarity (ANI >91%) to porcine-derived strains. This was supported by whole-genome SNP (wgSNP) analysis, whereby the ST138 bovine-derived isolates formed a clonal lineage and displayed a diverse population structure compared to porcine strains. Resistome profiling uncovered antimicrobial resistance gene (ARG) content, bovine isolates reflecting only four core ARGs i.e., *dfrC, mgrA, norA*, and *tet(38)*, which confer resistance to trimethoprim, fluoroquinolones, and tetracyclines. In contrast, the compared porcine strains harboured a diverse set of resistance determinants, including *blaZ, ermC, tet(K)*, and *vgaALC* that encode for beta-lactams, macrolides, tetracycline, and lincosamides, respectively. The five *S. chromogenes* isolates grouped into two 2 sequence types, namely ST138 and ST62. Pangenome reconstruction of 177 global genomes confirmed that *S. chromogenes* possesses an open pangenome, with only ~17.5% of genes conserved as core or soft-core elements. Notably, unique strain-specific genes of the ST138 were determined to be associated with trehalose metabolism identified in bovine isolates, potentially reflecting niche-specific adaptation to the mammary environment in the Free State Province of South Africa.

**Conclusion:**

These findings advance our understanding of *S. chromogenes* population structure and resistance ecology. They underscore the importance of continued genomic surveillance of livestock pathogens to inform targeted intervention strategies and improve animal health in diverse production settings, and further clarify the implications for future antibiotic therapy and prevention of infections associated with this species.

## 1 Introduction

Worldwide, subclinical mastitis (SCM) is the most common disease in dairy farming and a major financial burden (Hoque et al., 2019). Although injury to the mammary gland typically results in a marked reduction in milk supply, the identification of mastitis is compromised in the absence of clinical indicators (Simojoki et al., 2009; De Buck et al., 2021). Since *Staphylococcus* species are believed to be the primary agents that cause mastitis and SCM, it is important to note that the contagious *S. aureus* and other species of coagulase-negative staphylococci (CoNS), such as *S. haemolyticus, S. chromogenes, S. epidermidis, S. sciuri*, and *S. simulans*, are frequently isolated from bovine SCM cases (De Visscher et al., 2017).

More than 20 non-aureus staphylococci (NAS) species have been linked to mastitis and bovine intramammary infection (IMI) (De Buck et al., 2021). Among these, one of the most common species in subclinical mastitis is *S. chromogenes* (Nyman et al., 2018; De Buck et al., 2021; Waller et al., 2023). To our knowledge, few studies has employed multilocus sequence typing (MLST), which can enhance the distinction of strains and subspecies, out of a few that have used various molecular techniques In a study by Waller et al (Waller et al., 2023), they identified and differentiated between 105 *S. chromogenes* isolates using MLST and their results revealed ST19, ST102, and ST103 were the most abundant sequencing types detected. Furthermore, (Huebner et al., 2021). also detected 46 sequence types amongst 102 *S. chromogenes* isolates, of which ST1 was the most common sequence type observed in the sample population followed by ST6 and ST18 and ST15, ST28, ST15 and ST5, respectively. When compared to IMI caused by *Staphylococcus aureus*, NAS IMI typically causes less severe symptoms and fewer long-term effects. A noticeable increase in milk somatic cell counts (SCC) and chronic IMI, which may be caused by *S. chromogenes*, could negatively impact dairy farmers’ economy (Nyman et al., 2018; De Buck et al., 2021). Besides the species’ genotypic diversity, the therapeutic effects of S. chromogenes IMI differ from study to study.

Research on *S. aureus* related to cattle has revealed both genotypic and virulence gene occurrence variation among isolates (Lundberg et al., 2014; Huebner et al., 2021). However, little is known about the genotypic variation of these bacteria, which limits our understanding of the epidemiology of bovine *S. chromogenes* IMI. T.

Whole-genome sequencing (WGS) can also assist in shedding light on the epidemiology and genomic variation of these bacteria as well as the existence and spread of possible clones or clonal lineages (Lekota et al., 2020; Thomas et al., 2021). Additionally, WGS can reveal whether bovine *S. chromogenes* has genes encoding antimicrobial resistance (AMR) or putative virulence factors (pVF). While a few studies from other countries have used WGS to describe variations in the occurrence of known virulence factors (VF) among NAS (Naushad et al., 2016; Ocloo et al., 2024) these studies did not perform sequence typing, and pVF and genes encoding AMR in *S. chromogenes* have not been identified in any previous studies conducted in South Africa using WGS (Fergestad et al., 2021; Camargo et al., 2022).

Comparative genomics offers a strong framework for exploring intra-species diversity, uncovering strain-level evolutionary relationships, and identifying genetic factors related to antimicrobial resistance (AMR) and virulence (Montelongo et al., 2022). While the availability of WGS data for non-aureus staphylococci (NAS) has increased, studies specifically focusing on the comparative genomics of *S. chromogenes* are still limited. This is particularly true in low- and middle-income countries like South Africa, where regional farming practices, selective antibiotic use, and environmental factors may lead to unique genomic adaptations (Sheppard et al., 2018; Ajose et al., 2024).

To date, the application of advanced comparative genomics tools, such as pangenome analysis and whole-genome-based single-nucleotide polymorphism (wgSNP) analysis, has not been thoroughly explored for *S. chromogenes* in South Africa. These methods are crucial for accurately placing newly sequenced isolates within both global and local population structures (Magome et al., 2025). They also help identify conserved and accessory genome components that may contribute to host adaptation, persistence, or pathogenic potential (Sheppard et al., 2018).

Moreover, the presence and diversity of antimicrobial resistance genes (ARGs) and virulence-associated factors in *S. chromogenes*, especially those linked to bovine intramammary infections, remain largely unexplored. While several studies have addressed NAS species in general, there exists a significant knowledge gap regarding *S. chromogenes* isolates from subclinical mastitis cases in dairy cattle. This gap is especially pronounced in South Africa, where, to our knowledge, no bovine-derived *S. chromogenes* genomes have been described in the literature or submitted to public databases. Up to date, only a small number of genomes (n = 12), all from porcine sources in South Africa, are available in GenBank (Ocloo et al., 2024). In this study, we performed whole-genome sequencing and comparative genomic analysis on five *S. chromogenes* isolates recovered from cases of subclinical mastitis in South African dairy herds. We employed wgSNP-based phylogenetic reconstruction alongside pangenome analysis to assess the global genetic diversity, evolutionary relationships, and potential clonal structure of these isolates. We also characterized their resistome and virulome profiles to gain insights into the genomic features contributing to pathogenesis and antimicrobial resistance. To our knowledge, this represents the first genome-based study of bovine *S. chromogenes* in South Africa, providing foundational data that enhances our understanding of this emerging mastitis pathogen in the region.

## 2 Materials and methods

### 2.1 Sample selection and sample collection

The five selected genomes form part of the bovine mastitis surveillance collected from our previous study (Khasapane et al., 2024). Briefly, the five strains were isolated from 55 milk samples from bovine subclinical mastitis cases collected in seven small-scale farms in three local Municipalities (Mantsopa (Latitude: 29.1283°S, Longitude: 27.2676°E), Setsoto (Latitude: 28.5302°S, Longitude: 27.6435°E), and Maluti-a-Phofung (Latitude: 28°16′21.94′′S) in the Free State Province of South Africa between year 2021–2022.

### 2.2 Microbiological and molecular tests

Following the guidelines set forth by the National Mastitis Council (2004), milk samples were collected. Using the somatic cell count (SCC) method and flow cytometry, 166 composite milk samples from individual cows were chosen at random and examined for intramammary infection (Mérieux NutriSciences, South Africa). According to the recommendations of Karzis et al (Karzis et al., 2017), the CMT results were scored and interpreted as follows: 0 (negative [healthy quarter], somatic cell count [SCC] ≤100,000 cells/ml milk), 1+ (weak positive, SCC >100,000 < 500,000 cells/ml milk), 2+ (distinct positive, SCC >500,000 < 1000,000 cells/ml milk), and 3+; (strong positive, SCC ≥1000,000 cells/ml milk).Gram staining and the RapID STAPH PLUS kit were used to presumptively identify the *Staphylococcus chromogenes* isolates as Gram-positive cocci. In accordance with Ozbey et al (Ozbey et al., 2022), confirmation was obtained by means of PCR and matrix-assisted laser desorption ionization–time-of-flight mass spectrometry (MALDI-TOF MS). Disk diffusion were used for phenotypic antimicrobial resistance profiling, respectively (Khasapane et al., 2024). The recommendations established by the Clinical Laboratory Standards Institute were adhered to in the testing of antibiotic susceptibility (CLSI (Clinical and Laboratory Standards Institute) and CLSI, 2023). Ten micrograms of gentamicin, ten micrograms of ampicillin, thirty micrograms of tetracycline, ten micrograms of penicillin, fifteen micrograms of erythromycin, five micrograms of ciprofloxacin, and fifteen micrograms of cefoxitin were distributed using antibiotic disks (ThermoFischer, South Africa). The results were classified using CLSI criteria as resistant (R), intermediate (I), or sensitive (S).

### 2.3 Genomic extractions

Quick-DNA™ Fungal/Bacterial Miniprep Kit was used to extract genomic DNA from respective isolates according to the manufacturer’s instructions (Zymo Research, CA 92614, USA). The quantity of gDNA extracted from samples was measured using the Qubit 2.0 fluorometer (ThermoFisher, USA).

### 2.4 Whole-genome sequencing

Whole-genome sequencing of the five *Staphylococcus chromogenes* isolates was conducted at the Sequencing Core Facility of the National Institute for Communicable Diseases (NICD), South Africa. Library preparation was carried out using the Illumina DNA Prep kit (Illumina, San Diego, CA, USA), following the manufacturer’s protocol. Briefly, DNA libraries were constructed with multiplexed dual indices, enabling paired-end sequencing (2 × 150 bp). The prepared libraries were quantified, normalized, and pooled prior to sequencing. Sequencing was performed on the Illumina NextSeq 2000 platform (Illumina, San Diego, CA, USA).

### 2.5 Quality control and *de novo* assembly

*De novo* assembly was performed using SKESA v2.3.0 (Souvorov et al., 2018), and the assemblies were optimized using Shovill v1.1.0 https://github.com/tseemann/shovill, with depth and minimum contig length set to 100 and 200, respectively. Assembly metrics were assessed using QUAST v5.0.2 (Gurevich et al., 2013). The completeness and contamination estimate of the genomes was evaluated using CheckM (Parks et al., 2015; Li et al., 2023). Assembly statistics, such as N50, total length, and GC content, were calculated using QUAST v2.3 (Gurevich et al., 2013). The taxonomic classification of bacterial strains was carried out *in silico* using the Pasteur database integrated into PathogenWatch (https://pathogen.watch/) and MLST (Li et al., 2023). In accordance with the methods described by Chaumeil et al (Chaumeil et al., 2020), *Staphylococcus* species were identified using GTDBtk v1.7.0.

### 2.6 Genomic comparison based on average nucleotide identity

To investigate the genomic relatedness among *S. chromogenes* isolates from bovine and porcine sources, we calculated pairwise average nucleotide identity (ANI) values using the pyANI tool v0.2.12 within the Anvi’o platform v8.0.1 (Arkin et al., 2018). The analysis included a total of 17 genomes: five isolates (SC12, SC14, SC21, SC28, and SC33) from bovine mastitis cases and twelve publicly available porcine-associated isolates (**Supplementary Table 1**). All genomes were formatted and processed according to the Anvi’o pangenomics workflow, removing contigs less than 200bp. Specifically, each genome assembly was converted into Anvi’o contig databases using the anvi-gen-contigs-database command, followed by functional annotation using anvi-run-hmms. We computed the ANI using the anvi-compute-genome-similarity command, which wraps pyANI and applies BLAST-based ANI calculations (ANIb method) across all genome pairs. The analysis was conducted with default parameters, and the results were visualized as an ANI matrix and hierarchical clustering dendrogram. The resulting ANI values were interpreted using a species delineation threshold of 95% identity. Since the 95% average nucleotide identity (ANI) threshold often corresponds with the traditional DNA-DNA hybridization (DDH) method, which was once regarded as the gold standard for species identification, it is frequently used to differentiate between bacterial and archaeal species. When two genomes have ANI values above 95%, they generally belong to the same species; if they are below this threshold, they may represent separate species (Eren et al., 2021).

### 2.7 Antimicrobial resistance and virulence factors predictions

Antibiotic resistance and virulence genes in the five *S. chromogenes* genomes were screened using the ABRicate pipeline [accessed on June, 2025] and AMRFinderplus (Eren et al., 2021). We included 12 South African available *S. chromogenes* in GenBank for comparison of the ARGs, VF and plasmid replicon genes. The Comprehensive Antibiotic Resistance Database (CARD) database (Feldgarden et al., 2022) was used to identify antimicrobial resistance determinants in the assembled genome. Minimum identity and coverage requirements of 70% (–minid 70) and 70% (–mincov 70) were applied, respectively. Additionally, antibiotic resistance genes were found using the CARD database. Utilizing the Virulence Factor Database (Hackenberger et al., 2025), ABRicate was also utilized to assess virulence factors and efflux pump coding genes in the sequenced genome. The minimum identity and coverage requirements were 70% (–minid 70) and 70% (–mincov 70), respectively. Additionally, ABRicate used the Plasmid Finder database (https://www.genomicepidemiology.org/) to identify plasmid replicons in the genomes that were sequenced. Chiplot (https://www.chiplot.online/accessed on 25 June 2025) was used to visualize the resistance profiles.

### 2.8 Pangenome and single-nucleotide polymorphism whole-genome analysis

A total of 177 *S. chromogenes* genomes were analyzed for pangenomic profiling, comprising 172 publicly available genomes retrieved from GenBank (**Supplementary Table 1**) and five newly sequenced genomes generated in this study. Genome selection was based on the availability of *S. chromogenes* assemblies in GenBank at the time of analysis. The dataset included isolates from Canada (n = 84), China (n = 26), Brazil (n = 9), India (n = 11), Finland (n = 8), Belgium (n = 4), Thailand (n = 5), South Africa (n = 12), Norway (n = 2), Somalia (n = 2), the United States of America (n = 3), and two isolates of unknown origin. Among these, 110 genomes were derived from cases of bovine mastitis. All genomes, including the five newly sequenced ones, were annotated using Prokka v1.14 (Liu et al., 2022). Similarity searches among coding DNA sequences (CDSs) were performed using pairwise BLASTp, and orthologous gene clusters were identified using the Markov Cluster Algorithm (MCL). Pangenome clusters were classified as core genes (present in 100% of genomes), soft core genes (present in ≥95% of genomes), shell genes (present in 15–95% of genomes), and cloud genes (present in <15% of genomes). Core genome alignments were used to extract single nucleotide polymorphisms (SNPs) with SNP-sites v2.5.1 (https://github.com/sanger-pathogens/snp-sites), and recombination events were detected and removed using Gubbins v3.4 (Eren et al., 2021). A maximum likelihood phylogenetic tree was constructed from the filtered core SNPs using IQ-TREE v1.6.10 (Seemann, 2014). Pan-genome presence/absence matrices were visualized in RStudio using the pheatmap package to generate heatmaps of gene distribution across isolates.

## 3 Results

### 3.1 Phenotypic antimicrobial profiles

As reported in our previous study, the *S. chromogenes* isolates showed resistance to penicillin (100%), followed by ampicillin (40%), gentamycin (40%), ciprofloxacin (100%), and cefoxitin (40%), respectively (Sheppard et al., 2018). Of these isolates, SC1_2 was resistant to penicillin, ampicillin, and ciprofloxacin, while SC1_4 and SC3_8 were resistant to penicillin, gentamycin, and ciprofloxacin. Whereas SC2_1 was resistant to penicillin, ampicillin, ciprofloxacin, and cefoxitin, while SC2_8 was resistant to penicillin, ciprofloxacin, and cefoxitin.

### 3.1 Whole genome sequencing of *S. chromogenes*

In this study, the whole genomes of five *S. chromogenes* isolates linked to bovine subclinical mastitis from smallholder farms in the Free State Province were sequenced. **Table 1** provides an overview of genomic metrics of five bovine-associated strains sequenced in this study (SC1_2, SC1_4, SC2_1, SC2_8, and SC3_3) and twelve publicly available porcine-derived genomes (ST150 to ST161) from South Africa. The total genome sizes ranged from 2.27 Mb (ST150) to 2.43 Mb (ST161), with bovine-derived genomes averaging 2.35 Mb in length. All genomes had a GC content consistent with previous *S. chromogenes* reports (~36%). The number of contigs in the bovine isolates ranged from 13 to 29, with SC1_2 showing the most contiguous assembly (13 contigs, L50 = 1). The longest contig sizes for these isolates ranged from 324,307 bp (SC33) to 1,185,030 bp (SC12). In contrast, porcine genomes had between 14 and 29 contigs, with the longest contig reaching 1.34 Mb in ST161. N50 values among bovine genomes varied from 180,129 bp (SC2_1) to 1,185,030 bp (SC1_2), reflecting differences in assembly contiguity. Gene prediction using Prodigal identified between 2,229 and 2,415 protein-coding genes across all isolates. The bovine genomes encoded between 2,296 and 2,352 genes, aligning closely with the porcine genomes, which had 2,227–2,325 predicted genes. All bovine-derived isolates encoded 55–57 tRNAs, higher than the 38–43 tRNAs observed in porcine genomes.

**Table 1:** Genomic statistics of the five sequenced *S. chromogenes* genomes with the compared 12 porcine genomes from South Africa.

Contigs stats	SC1_2*	SC1_4*	SC2_1*	SC2_8*	SC3_3*	ST150	ST151	ST152	ST153	ST154	ST155	ST156	ST157	ST158	ST159	ST160	ST161
Total Length	2,351,579	2,364,766	2,352,423	2,351,698	2,352,215	2,272,030	2,343,140	2,265,120	2,318,088	2,364,429	2,342,193	2,337,974	2,296,366	2,352,050	2,337,691	2,307,655	2,434,252
Num Contigs	13	19	25	28	29	25	21	19	26	29	25	14	26	22	22	21	18
Num Contigs > 100 kb	4	5	8	7	9	6	7	8	7	7	6	5	8	6	7	6	5
Num Contigs > 50 kb	6	9	11	13	13	9	9	10	12	11	8	7	9	7	9	10	6
Num Contigs > 20 kb	11	13	15	16	19	13	11	11	15	14	9	9	10	11	11	13	11
Num Contigs > 10 kb	11	13	16	17	20	14	11	11	18	17	12	10	12	12	11	13	11
Num Contigs > 5 kb	11	13	16	17	20	14	13	11	18	17	12	10	13	13	13	14	12
Longest Contig	1,185,030	1,068,224	574,405	413,053	324,307	875,479	702,652	700,425	628,426	653,473	648,688	706,191	516,936	802,328	701,891	693,465	1,339,295
Shortest Contig	629	430	392	385	358	507	514	548	605	605	512	521	533	596	514	514	560
Num Genes (prodigal)	2,296	2,352	2,311	2,318	2,311	2,229	2,286	2,223	2,264	2,323	2,31	2,29	2,27	2,325	2,279	2,255	2,415
L50	1	2	4	4	5	2	2	3	3	3	3	2	3	2	2	2	1
L75	3	5	7	8	9	5	5	5	8	7	5	4	6	5	5	5	3
L90	6	8	11	12	14	8	8	8	11	11	7	6	8	7	8	9	5
N50	1,185,030	249,622	180,129	278,28	180,571	297,155	533,686	249,37	248,943	276,534	307,562	538,126	331,844	378,096	533,687	520,712	1,339,295
N75	288,484	190,494	140,06	94,977	103,932	134,047	129,289	129,341	96,934	119,423	249,229	258,011	195,383	249,652	129,289	126,909	249,536
N90	85,45	85,775	64,69	87,811	49,988	78,699	88,766	113,273	84,997	71,092	95,305	88,766	113,191	88,764	88,766	78,703	114,833
Transfer_RNAs	55	55	57	57	57	39	38	43	39	39	39	39	39	38	41	42	38
MLST	62	62	138	138	138	158	150	151	152	154	155	156	157	158	169	160	161

*Indicate genomes sequenced in this study derived from bovine mastitis.

### 3.2 Genomic relatedness of the South African *Staphylococcus chromogenes* isolates based on ANI

**Figure 1:**
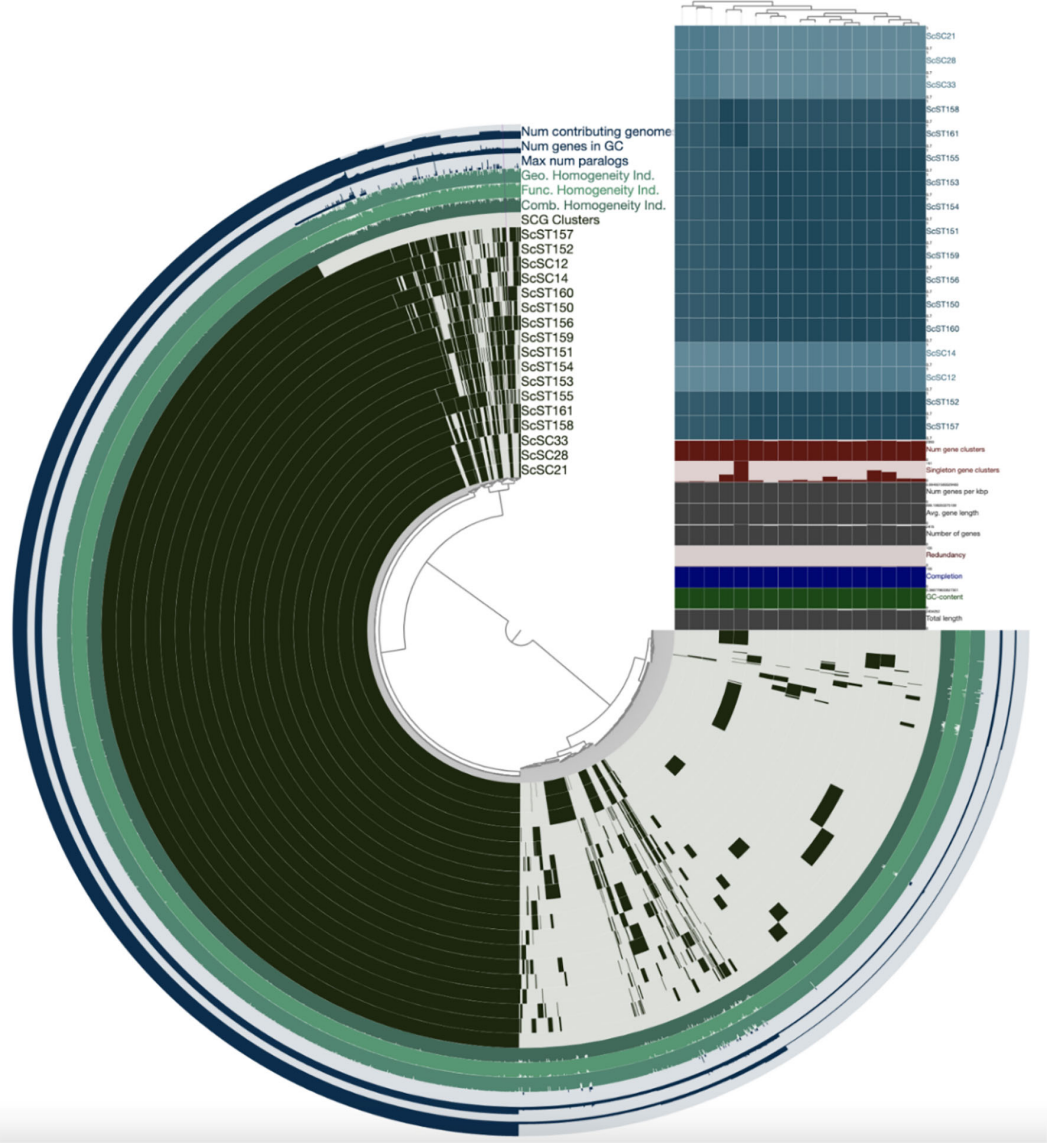
Visualization of pangenome analyses carried out by ANVI’O, showing the average nucleotide identity clustering among the 5 *Staphylococcus chromogens* isolated from bovine subclinical mastitis and 12 porcine isolates retrieved from GenBank.

To assess the genomic similarity between *S. chromogenes* isolates recovered from bovine mastitis and porcine sources available in GenBank from South Africa, pairwise average nucleotide identity (ANI) values were computed among all 17 genomes (**Figure 1**). In contrast, SC1_2 and SC1_4 showed lower ANI values with SC2_1–SC3_3 (85.6%–86.1%) and each other (93.4%), suggesting greater genetic divergence within the bovine group. When comparing bovine and porcine isolates, SC1_2 and SC1_4 shared higher ANI values (>91%) with certain porcine isolates than with the SC2_1–SC3_3 group. For instance, SC1_2 exhibited the highest ANI with ST150 (95.2%), followed by ST152 (94.5%) and ST153 (91.9%). Similarly, SC1_4 displayed the greatest similarity to ST152 (95.0%) and ST157 (93.5%). These values indicate that SC1_2 and SC1_4 are more closely related to selected porcine strains than to other bovine isolates from this study. In contrast, SC2_1, SC2_8, and SC3_3 consistently showed lower ANI values (~84%–86%) with all porcine isolates, supporting the notion that these strains are more distantly related and may represent a bovine-adapted lineage. The most divergent porcine strain in this comparison was ST161, which showed 82.0%–84.2% ANI values with bovine isolates, falling well below the 95% species delineation threshold.

### 3.3 Virulence and resistome analysis of the South African *S. chromogenes* genomes

**Figure 2:**
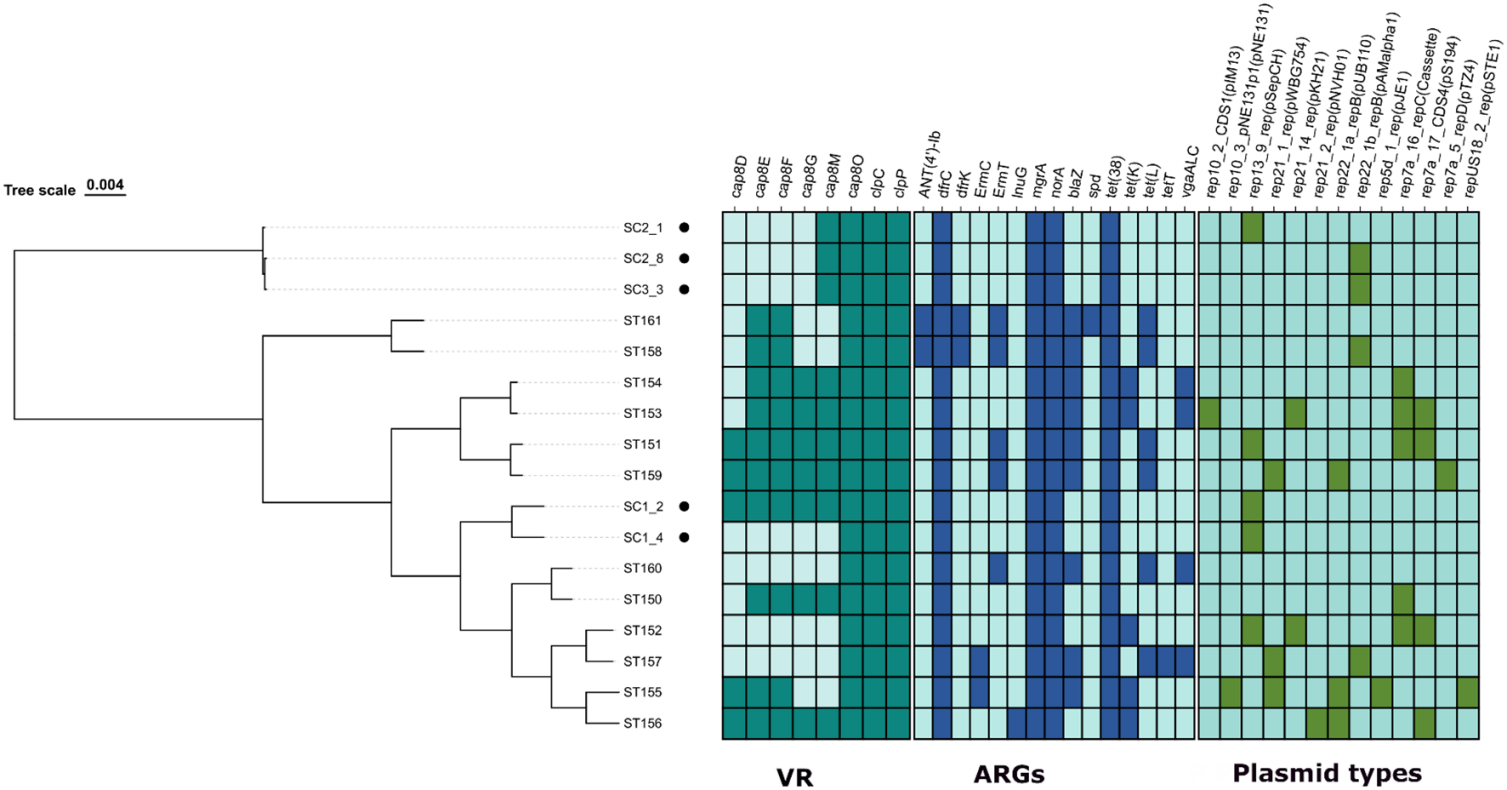
Resistome and virulence profiling of the 5 *Staphylococcus chromogens* isolated from bovine subclinical mastitis and 12 porcine isolates retrieved from GenBank.

To determine whether the isolates had any resistance genes, a comprehensive analysis of the genomes of 17 *S. chromogenes* was conducted (**Figure 2**). The analysis identified eight virulence-associated genes: six involved in capsular polysaccharide biosynthesis (*cap8D, cap8E, cap8F, cap8G, cap8M, cap8O*) and two related to stress response (*clpC, clpP*) across the genomes compared. Among all 17 genomes, *cap8O, clpC*, and *clpP* were found in every strain, indicating their potential role as core virulence or fitness factors in *S. chromogenes*. Conversely, the other *cap8* genes showed variable presence among the isolates. Of the bovine-derived isolates, only SC1_2 strain contained a complete cap8 operon (*cap8D–cap8O*). The other four bovine strains were missing the upstream capsular genes (*cap8D–cap8G*) but retained *cap8M* and *cap8O*, indicating partial conservation of the capsule biosynthesis locus in their genomes. The porcine-derived isolates displayed more diversity in the *cap8* gene cluster. Three isolates (ST151, ST156, and ST159) possessed the entire cap8 operon, while the others had partial gene content. For example, isolates like ST153, ST154, ST158, and ST161 retained combinations of *cap8E, cap8F, cap8G*, and *cap8M* genes. Notably, three porcine isolates (ST152, ST157, and ST160) lacked all *cap8* genes except for *cap8O*.

A total of 15 antimicrobial resistance genes (ARGs) were identified across the 17 *S. chromogenes* genomes analyzed, comprising five bovine-derived isolates (SC1_2, SC1_4, SC2_1, SC2_8, and SC3_3) and twelve porcine-derived strains. Each genome contained four core ARGs: *dfrC, mgrA, norA*, and *tet(38)*, which confer resistance to trimethoprim, fluoroquinolones, and tetracyclines. Interestingly, these were the sole ARGs found in all five isolates derived from cattle, suggesting a relatively conserved and limited resistance profile in strains linked to mastitis. Conversely, the isolates from pigs displayed a wider and more varied array of ARGs. The *blaZ* gene, responsible for encoding beta-lactamase, was found in 10 out of the 12 porcine genomes (83%) but was absent in all bovine-derived strains. Additional tetracycline resistance genes, *tet(K)* and *tet(L)*, were exclusively identified in porcine isolates, with *tet(K)* present in seven and *tet(L)* in six genomes. The *tetT* gene appeared in only one porcine strain (ST157). Resistance genes related to macrolide-lincosamide-streptogramin (MLS) antibiotics, such as *ermC, ermT*, and *lnuG*, were also confined to porcine isolates. The aminoglycoside resistance gene *ANT(4’)-Ib* was detected in two porcine genomes (ST158 and ST161), and the streptogramin resistance gene *vgaALC* was found in the ST153, ST154, and ST160 porcine genomes. Porcine strains ST158, ST160, and ST161 exhibited the highest number of ARGs, each containing eight or more resistance determinants. These findings highlight a distinct difference in ARG distribution based on host origin, with porcine-derived *S. chromogenes* strains showing a greater variety and number of resistance genes, likely due to different antimicrobial selection pressures in swine production environments compared to those in the bovine dairy setting. Across the 17 Staphylococcus chromogenes genomes analyzed, a total of 14 unique plasmid replicon types were identified. These replicons included members of various plasmid families, such as rep10_2_CDS1(pIM13), rep13_9_rep(pSepCH), rep21_1_rep(pWBG754), rep21_14_rep(pKH21), rep21_2_rep(pNVH01), rep22_1a_repB(pUB110), rep22_1b_repB(pAMalpha1), rep5d_1_rep(pJE1), rep7a_16_repC (Cassette), rep7a_17_CDS4(pS194), rep7a_5_repD(pTZ4), rep10_3_pNE131p1(pNE131), and repUS18_2_rep(pSTE1).

Among the five isolates derived from cattle, only two types of plasmid replicons were identified: rep13_9_rep(pSepCH) found in SC1_2, SC1_4, and SC2_1, and rep22_1b_repB(pAMalpha1) present in SC2_8 and SC3_3. This indicates a limited variety of plasmids in bovine isolates. Conversely, plasmid replicons were more commonly found and varied in isolates from pigs. Strains ST153 and ST155 showed the greatest diversity, each harboring up to four distinct plasmid types. Interestingly, rep7a_16_repC (cassette) and rep7a_17_CDS4(pS194) were often detected together in several porcine isolates, such as ST151, ST152, and ST153, suggesting a possible connection or shared plasmid structures. Some replicons seemed to be associated with specific hosts, with rep13_9_rep(pSepCH) being more common in bovine isolates, while others, like rep7a_16_repC, rep22_1a_repB, and rep21_1_rep, were mostly found in porcine strains.

### 3.4 Pangenomes analysis among 177 global *S. chromogenes* genomes

Pangenome analysis of the global 177 *Staphylococcus chromogenes* revealed a highly diverse genomic landscape, comprising 9,063 non-redundant genes across all analyzed strains (**Supplementary Figure S1**). These genes were categorized based on their frequency across strains, reflecting varying levels of conservation and variability within the species. A total of 1,265 genes were identified as core genes, present in at least 99% of strains. These genes are presumed to encode essential cellular functions, including fundamental processes such as replication, transcription, translation, and primary metabolism, forming the conserved genomic backbone of *S. chromogenes*. An additional 320 genes were classified as soft-core genes, present in 95–98% of strains, suggesting that while nearly universal, these genes may be dispensable under specific conditions or lost in a small subset of strains. The accessory genome, comprising genes with variable presence, was substantial. Shell genes, found in 15–94% of strains, accounted for 1,009 genes. These likely include genes involved in environmental adaptation, niche specialization, stress response, and potentially strain-specific virulence or resistance mechanisms. Strikingly, the majority of the pangenome’s 6,469 genes were classified as cloud genes, present in fewer than 15% of strains. Together, the core and soft-core genome made up only 17.5% (1,585 genes) of the total pangenome, while the remaining 82.5% (7,478 genes) constituted the accessory genome (shell and cloud genes).

### 3.5 Whole genome single nucleotide polymorphism analysis

**Figure 3:**
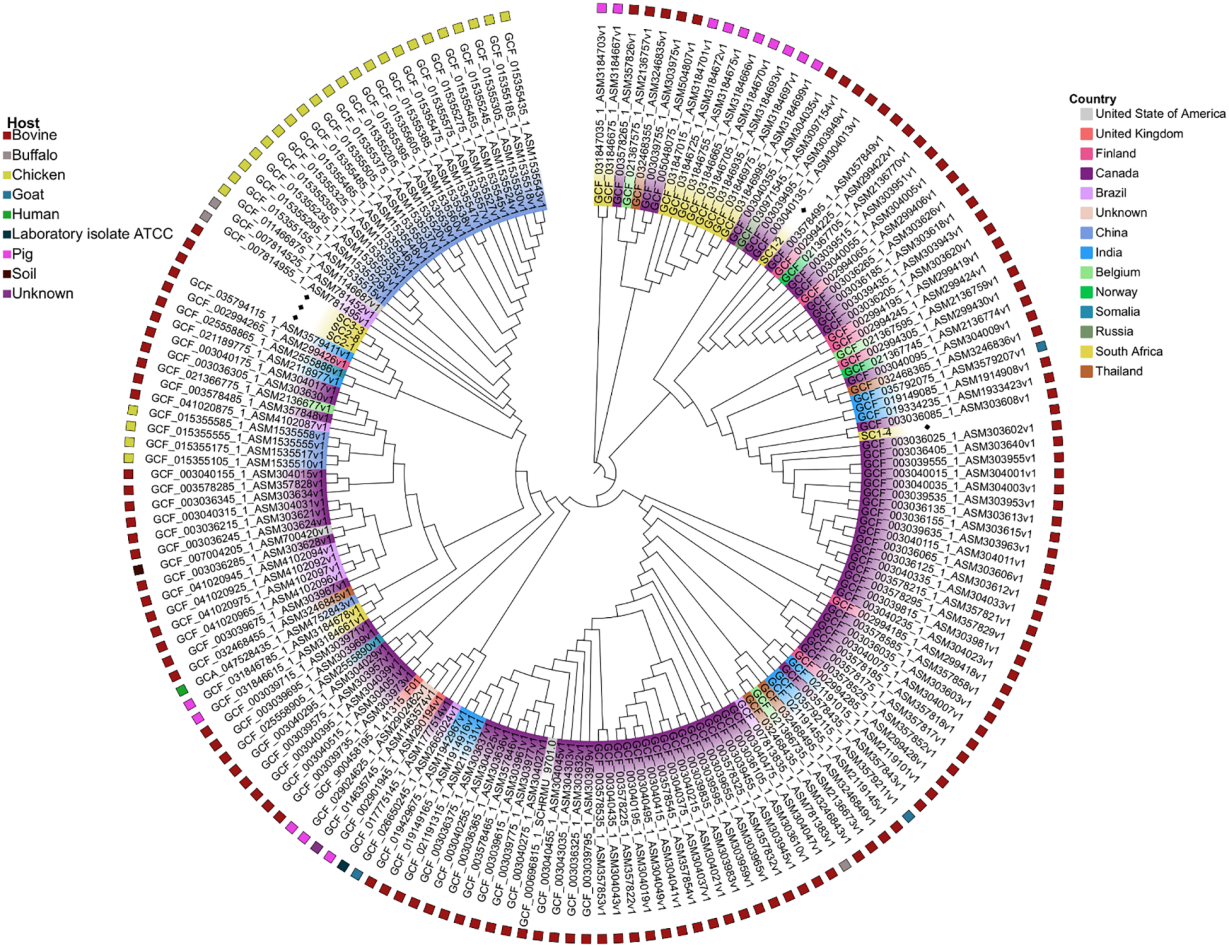
Whole-genome single-nucleotide polymorphism phylogenetic analysis of the 177 *Staphylococcus chromogenes* based on maximum likelihood. The leaves on the branches are colored based on the country of origin, while the outer ring presents the hosts.

Based on whole-genome single-nucleotide polymorphism (wgSNP) phylogenetic analysis, five major SNP lineages were observed (**Figure 3**). The sequenced SC1–4 strain clustered with the Canadian strain SNUC 4584 (GenBank accession GCF_003036025.1), while SC1–2 appeared to be a distinct isolate based on its SNP profile, forming a separate cluster. In contrast, the three strains SC3_3, SC2_1, and SC2_8 grouped and clustered closely with bovine milk-derived strains from India (GCF_035794115.1) and Finland (GCF_002994265.1). Notably, this study presents the first *S. chromogenes* genomes derived from bovine mastitis cases in South Africa, as all previously reported South African genomes (n = 12) were isolated from pigs.

## 4 Discussion

In this study, *S. chromogenes* isolates from bovine subclinical mastitis cases in smallholder dairy farms in the Free State Province of South Africa were examined for antibiotic resistance patterns and virulence gene traits, as well as the global population structure to place the sequenced genomes. This study presents the first comparative genomic analysis of *S. chromogenes* isolates associated with bovine mastitis in South Africa, providing insights into strain diversity, antimicrobial resistance, virulence potential, and plasmid content, with further contextualization against porcine-derived genomes from GenBank. Our findings underscore the genomic heterogeneity within *S. chromogenes*. Pairwise ANI analysis revealed clear clustering patterns among the bovine isolates, with SC2_1, SC2_8, and SC3_3 forming a tight cluster (>99.7% ANI), indicative of clonal or near-clonal relatedness and likely representing a bovine-adapted lineage (ST138). In contrast, isolates SC1_2 and SC1_4 were genomically divergent both from the ST138 group and from each other, sharing higher ANI values (>91%) with specific porcine strains. This suggests either historical host-switching events or the circulation of shared lineages across host species. Such findings echo reports from other *Staphylococcus* species, where host-specific adaptation is often accompanied by genomic divergence and selective acquisition or loss of mobile elements and virulence traits (Nguyen et al., 2015).

The sequenced isolates were categorized into sequence types ST138 and ST62 by *in-silico* MLST analysis, showing clonal and diverse strains. This is consistent with previous research that found *S. chromogenes* infections in cows (Becker et al., 2014). However, our results were also inconsistent with (Waller et al., 2023). and (Huebner et al., 2021). who have identified ST19, ST102, ST103, ST1 ST6, ST18, ST15, ST28, ST15 and ST5, respectively. The virulence gene analysis revealed a core set of fitness- and capsule-associated genes across the 17 *S. chromogenes* genomes, with notable variation in gene content between bovine and porcine isolates. Across all strains, three genes *cap8O*, *clpC*, and *clpP* were universally present, suggesting that they play essential roles in the survival and adaptability of *S. chromogenes* across different host environments (Smith et al., 2005). The *clpC* and *clpP*, components of the ATP-dependent Clp protease system, are known to be crucial in stress tolerance, protein quality control, and virulence regulation in various staphylococcal species (Balasubramanian et al., 2017). Their universal presence across isolates implies a conserved mechanism supporting the pathogen’s persistence under host-associated stress conditions, such as immune pressure or oxidative damage (Park and Ronholm, 2021). In contrast, significant variability was observed in the *cap8* gene cluster responsible for capsular polysaccharide biosynthesis. Only one bovine-derived isolate (SC1_2) harboured the complete *cap8* operon (*cap8D–cap8O*), while the remaining four bovine isolates lacked key upstream genes, including *cap8D–cap8G*, and retained only *cap8M* and *cap8O* genes. This partial capsule gene content may suggest reduced or altered capsule formation in some bovine isolates, potentially influencing immune evasion or adhesion capabilities. The capsule is a known virulence factor in staphylococci, contributing to resistance against phagocytosis and aiding in biofilm formation (Frees et al., 2014).

The porcine-derived isolates exhibited a broader distribution of capsule genes, with three strains (ST151, ST156, and ST159) carrying the complete *cap8* operon and others displaying various partial combinations. Interestingly, a subset of porcine strains (e.g., ST152, ST157, ST160) retained only *cap8O*, indicating substantial variation even within the same host group. The greater diversity in capsule gene content among porcine isolates may correlate with a wider range of ecological niches or selective pressures present in swine production environments (Tewari and Dey, 2024; Boyle-Vavra et al., 2001). The differential conservation of virulence genes between bovine and porcine isolates suggests possible host-specific adaptations. The relatively conserved virulence gene profile among bovine strains could reflect specialization to the mammary gland environment, where limited but critical factors are sufficient for colonization and persistence (Petrocchi-Rilo et al., 2021). However, the greater diversity of virulence genes in pig isolates may offer greater adaptability to endure in a range of anatomical locations or under various environmental stresses, such as exposure to antibiotics (Qu et al., 2019). These findings highlight the importance of capsule biosynthesis and stress response systems as key determinants of *S. chromogenes* fitness. The observed variability, particularly in capsule gene content, may influence pathogenic potential, immune evasion strategies, and host specificity (Petrocchi-Rilo et al., 2021). These genomic traits could also serve as valuable markers for epidemiological tracking and for differentiating strains based on virulence potential, especially in clinical or subclinical mastitis surveillance (McCarthy and Lindsay, 2010).

The resistome analysis of 17 *S. chromogenes* genomes revealed clear distinctions in antimicrobial resistance gene (ARG) content between bovine-derived and porcine-derived isolates, underscoring the impact of host-specific environments and antimicrobial usage practices on the evolution of resistance traits in this species. All isolates, regardless of host origin, harboured a conserved set of four core resistance genes *dfrC*, *mgrA*, *norA*, and *tet(38)*, which confer resistance to trimethoprim, fluoroquinolones, and tetracyclines. Interestingly, our phenotypic and genotypic AMR showed co-occurrences of fluoroquinolone antibiotics in the form of ciprofloxacin and *mgrA* and *norA* genes; however, we could not detect any other genes related to our phenotypic AMR results. The presence of these genes suggests that *S. chromogenes* possesses a baseline level of resistance potentially essential for survival in diverse host environments (Smith et al., 2005). Specifically, *norA* encodes a multidrug efflux pump and is commonly implicated in fluoroquinolone resistance in staphylococci, while *mgrA* regulates multiple virulence and resistance determinants, highlighting the dual role of certain genes in both resistance and pathogenicity (Petrocchi-Rilo et al., 2021).

Beyond these core genes, notable differences emerged in the overall ARG diversity and abundance between host groups. The bovine isolates showed a limited and relatively conserved resistance profile, carrying only the four core genes. This suggests lower antimicrobial selection pressure in bovine mastitis environments, particularly in subclinical cases where antimicrobial interventions may be less frequent or more regulated (Wang et al., 2024). In contrast, porcine-derived isolates exhibited a broader and more diverse resistome, harboring additional genes conferring resistance to beta-lactams (*blaZ*), tetracyclines (*tet(K)*, *tet(L)*, *tetT*), macrolide-lincosamide-streptogramin (MLS) antibiotics (*ermC*, *ermT*, *lnuG*), aminoglycosides (*ANT(4’)-Ib*), and streptogramins (*vgaALC*). The *blaZ* gene, present in 83% of porcine isolates and absent from all bovine strains, indicates beta-lactamase-mediated resistance likely driven by routine use of penicillins and related antibiotics in swine production (Magome et al., 2025). *S. chromogenes* (and other coagulase-negative staphylococci, or CoNS) intrinsically carry the *tet(38)* gene, which encodes an efflux pump (Chen and Xie, 2019). This pump confers low-level resistance to tetracyclines and is conserved across many staphylococcal species, especially commensals or opportunistic pathogens. A study by Argudín et al (Tomanić et al., 2023), has shown that tetracycline resistance genes like *tet(K/L/M)* are commonly found in CoNS from pigs and poultry but are rarer in isolates from cattle, especially those not under intensive production. The presence of multiple antimicrobial resistance genes (ARGs) in individual porcine isolates, particularly ST158, ST160, and ST161, each harboring eight or more resistance determinants, suggests either cumulative adaptation under sustained antimicrobial exposure or the integration of diverse resistance mechanisms. This expanded resistome may enhance bacterial survival in antibiotic-rich environments and potentially contribute to the persistence or spread of multidrug-resistant (MDR) lineages in swine production systems (Magome et al., 2025).

Plasmid analysis further supported host-associated genomic variation. While bovine isolates harboured only two plasmid replicon types, porcine strains exhibited higher plasmid diversity, with some genomes carrying up to four replicon types. Among the five bovine-derived isolates, only two plasmid replicon types were detected: *rep13_9_rep(pSepCH)* and *rep22_1b_repB(pAMalpha1)*. These were each present in a subset of the bovine isolates, indicating a relatively narrow plasmid repertoire in *S. chromogenes* strains associated with bovine mastitis. The porcine isolates harboured a broader and more complex plasmid profile. Several strains, particularly ST153 and ST155, carried up to four distinct plasmid replicon types, illustrating a higher capacity for plasmid-mediated gene acquisition and exchange. Notably, certain replicons such as *rep7a_16_repC (Cassette)* and *rep7a_17_CDS4(pS194)* were frequently co-detected within the same genomes, suggesting possible co-localization or linkage on composite plasmids (Naushad et al., 2016; Argudín et al., 2017). These plasmids may facilitate the co-transfer of multiple adaptive traits, including antimicrobial resistance, stress tolerance, and niche-specific fitness factors (Becker et al., 2014). While *rep13_9_rep(pSepCH)* appeared to be more common in bovine isolates, most of the remaining replicons such as *rep21_1_rep(pWBG754)*, *rep22_1a_repB(pUB110)*, and *rep10_2_CDS1(pIM13)* were predominantly or exclusively found in porcine isolates. This suggests that certain plasmid backbones may be preferentially maintained or selected in particular host environments, possibly due to compatibility with the host genome or selective pressures related to antibiotic exposure and microbial competition.

The higher plasmid diversity observed in porcine isolates aligns with their richer resistome profiles, implying that plasmids may play a significant role in the acquisition and dissemination of antimicrobial resistance genes (Ocloo et al., 2024). Therefore, the plasmid content of porcine isolates may contribute to the emergence of multidrug-resistant lineages within pig production systems. These findings underscore the need for plasmid-focused surveillance in *S. chromogenes*, particularly in swine-associated strains, to monitor the mobility of resistance and virulence factors (Mencía-Ares et al., 2022). Further sequencing and functional analysis of these plasmids are warranted to elucidate their gene content, transfer mechanisms, and role in strain adaptation. In a broader One Health context, understanding the dynamics of plasmid-mediated gene flow in animal pathogens like *S. chromogenes* is critical for anticipating and mitigating the risks of ARG dissemination across animal, human, and environmental interfaces (Nanra et al., 2013; Castañeda-Barba et al., 2024).

A pangenome analysis showed that South African bovine isolates, including SC3-3, SC2-1, and SC2-8, possessed 67 unique accessory genes, most of which were annotated as hypothetical proteins. Two of these *treP_3* and *treP_4* are predicted to encode components of the trehalose-specific phosphotransferase system (PTS), suggesting a potential metabolic adaptation for carbohydrate utilization that may offer a selective advantage in specific environments (Nanra et al., 2013; Mencía-Ares et al., 2022). Phylogenomic analysis based on whole-genome SNPs placed SC1–4 closely with Canadian strain SNUC 4584, isolated from a bovine subclinical mastitis case. This clustering supports the notion of potential global lineage conservation or convergence among bovine-associated *S. chromogenes* strains (Schwarz et al., 2018; Watanabe et al., 2018). The South African bovine-derived isolates SC3-3, SC2-1, and SC2-8 of the ST138 form a well-supported monophyletic cluster in both whole-genome single-nucleotide polymorphism (wgSNP) and pangenome analyses. This cluster is not only distinct from local porcine isolates and other South African bovine strains like SC1-4 but also shows strong phylogenetic relatedness to global bovine milk-derived strains: GCF_035794115.1 from India and GCF_002994265.1 from Finland. This shared lineage suggests a possible global bovine-associated clade of *S. chromogenes* adapted to the milk environment (Feßler et al., 2018). Despite geographical separation, the close genetic similarity implies either convergent evolution under similar host-specific selective pressures (e.g., dairy-associated ecological niches) or a common ancestral origin among bovine-adapted strains. The open pangenome and diverse SNP-based phylogenetic placements underscore the need for continued genomic surveillance to track strain evolution and emerging pathogenic lineages in both livestock and broader One Health contexts. These results indicate that *S. chromogenes* possesses an open pangenome, characterized by extensive gene flux and diversity across strains. Further analysis of the accessory genome revealed 67 genes that were uniquely associated with the sequenced *S. chromogenes* strains SC3-3, SC2-1, and SC2-8. These strain-specific genes represent a subset of the cloud genome and may contribute to unique phenotypic or adaptive features distinguishing these strains from others in the dataset. Of the 67 unique genes, 65 were annotated as hypothetical proteins, indicating a lack of characterized function and underscoring the need for further functional validation and characterization. These hypothetical genes may represent novel functions or strain-specific adaptations not yet described in public databases. Notably, two of the unique genes, *treP_4* and *treP_3*, both appear to be associated with carbohydrate transport and metabolism. Specifically, these genes are predicted to encode components of the trehalose-specific phosphotransferase system (PTS), a sugar uptake mechanism involved in the transport and phosphorylation of trehalose. The presence of *treP_3* and *treP_4* suggests that these strains may have enhanced capabilities to utilize trehalose as a carbon source, potentially conferring a selective advantage in environments where this disaccharide is available. Interestingly, the sequenced strain SC1–4 was found to cluster closely with the Canadian strain SNUC 4584 (GCF_003036025.1) in the phylogenomic analysis. SNUC 4584 strain was originally isolated from *Bos taurus* diagnosed with subclinical mastitis, highlighting a potential link between these strains in terms of host association or pathogenic potential.

A key limitation of this study is the sample size, followed by the high proportion of strain-specific genes annotated as hypothetical proteins, particularly among the bovine mastitis-associated *S. chromogenes* strains within ST138. Of the 67 unique genes identified in isolates SC3-3, SC2-1, and SC2-8, 65 lacked functional annotation, significantly limiting our ability to interpret their biological roles. These hypothetical genes may represent novel virulence factors, metabolic functions, or niche-specific adaptations; however, their impact remains speculative in the absence of functional validation. Additionally, the study was constrained by the exclusive use of short-read sequencing technologies, which can fragment repetitive or complex genomic regions and impede the complete resolution of plasmids. This limits our understanding of the genomic context, structural organization, and potential mobility of antimicrobial resistance genes (ARGs) and virulence factors. The integration of long-read sequencing platforms (e.g., Oxford Nanopore or PacBio) in future studies would enable the assembly of complete plasmid sequences and more accurate mapping of MGEs. Such advancements are critical for dissecting the dynamics of gene transfer, plasmid-mediated resistance, and the evolution of host-adapted *S. chromogenes* lineages in livestock environments.

## 5 Conclusion

This study offers the first comprehensive comparative genomic analysis of *S. chromogens* isolates obtained from South African dairy cattle, in relation to local porcine strains and a more extensive global dataset. The genomic relatedness analysis, utilizing ANI and wgSNP approaches, revealed that bovine-derived isolates are categorized into distinct lineages. Notably, three strains (SC3–3, SC2–1, and SC2–8) formed a unique cluster, indicating the potential existence of a clade specifically adapted to cattle. Resistome profiling indicated that cattle-associated isolates possess a limited and conserved repertoire of ARGs compared to the more diverse and extensive ARG content observed in porcine isolates. This variation likely reflects the differing antimicrobial selection pressures experienced in different host environments. Additionally, the distribution of capsular biosynthesis and stress-response genes varied among strains, suggesting differences in their virulence potential and ecological fitness. Plasmid analysis indicated greater diversity in replicon types among porcine isolates, whereas the bovine strains were characterized by only two types of replicons, further reinforcing the notion of host-associated genomic compartmentalization. Pangenome analysis confirmed that *S. chromogenes* has an open genome structure, characterized by a small core genome and a substantial proportion of accessory and strain-specific genes. Interestingly, several bovine isolates contained unique gene content potentially linked to carbohydrate metabolism, which may provide adaptive advantages in the mammary niche. These findings underscore the distinct evolutionary trajectories influenced by host specificity and highlight the necessity for continued surveillance of *S. chromogenes*. Such efforts are crucial for better understanding its role in bovine mastitis and the dissemination of antimicrobial resistance within livestock systems. These results further highlight how important it is to implement focused antimicrobial stewardship initiatives and efficient management strategies in order to lessen the negative effects of SCM on dairy output and animal health. This entails using antibiotics sparingly, using optimal management practices, and routinely monitoring antimicrobial resistance. It’s also crucial to teach farmers good cleanliness habits and milking methods.

## Data Availability

The datasets presented in this study can be found in online repositories. The names of the repository/repositories and accession number(s) can be found in the article/**Supplementary Material**.
