# Supplementary material for: Population structure, resistome, and virulome of Staphylococcus chromogenes strains from milk of subclinical bovine mastitis in South Africa
Source: Front Cell Infect Microbiol. 2025 Aug 22;15:1654546. doi: 10.3389/fcimb.2025.1654546 (PMC12411441; doi:10.3389/fcimb.2025.1654546)
Supplement: Supplementary file 1 [file DataSheet1.docx]

**Population structure, resistome, and virulome of *Staphylococcus chromogenes* strains from milk of subclinical bovine mastitis in South Africa**

Khasapane N.G^1^, Nkhebenyane S.J^1^, Lekota K.E^2^, Thekisoe O^2^, Ramatla T.^1,2^

^1^ Centre for Applied Food Safety and Biotechnology, Department of Life Sciences, Central University of Technology, 1 Park Road, Bloemfontein, 9300, South Africa

^2^Unit for Environmental Sciences and Management, North-West University, Potchefstroom, 2531, South Africa

**Supplementary Table 1: Genomes of 172 *S. chromogenes* retrieved from GenBank used from comparative genomics in this study.**

| **Country** | **Host** | **Organism Qualifier** | **Assembly Name** | **Assembly Accession** | **Contig N50** | **Size** | **Submission Date** | **Gene Count** | **BioProject** | **BioSample** |
| --- | --- | --- | --- | --- | --- | --- | --- | --- | --- | --- |
| Canada | Bovine | strain: SNUC 335 | ASM357858v1 | GCF_003578585.1 | 56194 | 2227626 | 2018/09/20 | 2219 | PRJNA342349 | SAMN06172884 |
| Canada | Bovine | strain: S48 | ASM504807v1 | GCF_005048075.1 | 238895 | 2392851 | 2019/05/01 | 2389 | PRJNA533291 | SAMN11456258 |
| Finland | Bovine | strain: 101 | ASM299406v1 | GCF_002994065.1 | 275727 | 2310830 | 2018/03/12 | 2303 | PRJNA357351 | SAMN06141062 |
| Finland | Bovine | strain: 46 | ASM299418v1 | GCF_002994185.1 | 135665 | 2211516 | 2018/03/12 | 2195 | PRJNA357351 | SAMN06141059 |
| Finland | Bovine | strain: 92 | ASM299419v1 | GCF_002994195.1 | 286535 | 2296439 | 2018/03/12 | 2283 | PRJNA357351 | SAMN06141061 |
| Finland | Bovine | strain: 121 | ASM299422v1 | GCF_002994225.1 | 566271 | 2273453 | 2018/03/12 | 2256 | PRJNA357351 | SAMN06141063 |
| Finland | Bovine | strain: 117 | ASM299424v1 | GCF_002994245.1 | 286109 | 2272273 | 2018/03/12 | 2256 | PRJNA357351 | SAMN06141075 |
| Finland | Bovine | strain: 38 | ASM299426v1 | GCF_002994265.1 | 256283 | 2341593 | 2018/03/12 | 2318 | PRJNA357351 | SAMN06142918 |
| Finland | Bovine | strain: 72 | ASM299428v1 | GCF_002994285.1 | 222088 | 2322098 | 2018/03/12 | 2335 | PRJNA357351 | SAMN06142921 |
| Finland | Bovine | strain: 22 | ASM299430v1 | GCF_002994305.1 | 280267 | 2300754 | 2018/03/12 | 2294 | PRJNA357351 | SAMN06142920 |
| India | Bovine | strain: Sil RS 8 | ASM3579411v1 | GCF_035794115.1 | 243270 | 2361012 | 2024/01/17 | 2379 | PRJNA636233 | SAMN29049698 |
| Belgium | Bovine milk | strain: Ani-LG-096 | ASM2136673v1 | GCF_021366735.1 | 249419 | 2371815 | 2022/01/05 | 2374 | PRJNA609060 | SAMN19114585 |
| Belgium | Bovine milk | strain: Ani-LG-072 | ASM2136677v1 | GCF_021366775.1 | 293655 | 2310759 | 2022/01/05 | 2325 | PRJNA609060 | SAMN19114583 |
| Belgium | Bovine milk | strain: Ani-GT-038 | ASM2136757v1 | GCF_021367575.1 | 595257 | 2313898 | 2022/01/05 | 2294 | PRJNA609060 | SAMN19114542 |
| Belgium | Bovine milk | strain: Ani-GT-041 | ASM2136759v1 | GCF_021367595.1 | 594702 | 2349267 | 2022/01/05 | 2347 | PRJNA609060 | SAMN19114543 |
| Brazil | Bovine milk | strain: B15 | ASM4102087v1 | GCF_041020875.1 | 220832 | 2315140 | 2024/08/02 | 2307 | PRJNA1129503 | SAMN42156958 |
| Brazil | Bovine milk | strain: B9 | ASM4102092v1 | GCF_041020925.1 | 1203013 | 2317176 | 2024/08/02 | 2296 | PRJNA1129503 | SAMN42156952 |
| Brazil | Bovine milk | strain: B11 | ASM4102094v1 | GCF_041020945.1 | 290904 | 2480300 | 2024/08/02 | 2436 | PRJNA1129503 | SAMN42156954 |
| Brazil | Bovine milk | strain: B7 | ASM4102096v1 | GCF_041020965.1 | 1203011 | 2309686 | 2024/08/02 | 2288 | PRJNA1129503 | SAMN42156950 |
| Brazil | Bovine milk | strain: B8 | ASM4102097v1 | GCF_041020975.1 | 1203011 | 2309686 | 2024/08/02 | 2291 | PRJNA1129503 | SAMN42156951 |
| Canada | Bovine milk | strain: SNUC 4584 | ASM303602v1 | GCF_003036025.1 | 1189663 | 2306987 | 2018/04/05 | 2345 | PRJNA342349 | SAMN06172947 |
| Canada | Bovine milk | strain: SNUC 5997 | ASM303603v1 | GCF_003036035.1 | 81034 | 2278425 | 2018/04/05 | 2266 | PRJNA342349 | SAMN06172944 |
| Canada | Bovine milk | strain: SNUC 5084 | ASM303606v1 | GCF_003036065.1 | 42219 | 2309555 | 2018/04/05 | 2319 | PRJNA342349 | SAMN06172942 |
| Canada | Bovine milk | strain: SNUC 5978 | ASM303608v1 | GCF_003036085.1 | 55161 | 2345896 | 2018/04/05 | 2384 | PRJNA342349 | SAMN06172943 |
| Canada | Bovine milk | strain: SNUC 5079 | ASM303610v1 | GCF_003036105.1 | 75590 | 2370316 | 2018/04/05 | 2385 | PRJNA342349 | SAMN06172941 |
| Canada | Bovine milk | strain: SNUC 4619 | ASM303612v1 | GCF_003036125.1 | 39059 | 2327135 | 2018/04/05 | 2350 | PRJNA342349 | SAMN06172940 |
| Canada | Bovine milk | strain: SNUC 4199 | ASM303613v1 | GCF_003036135.1 | 61061 | 2231382 | 2018/04/05 | 2214 | PRJNA342349 | SAMN06172938 |
| Canada | Bovine milk | strain: SNUC 4192 | ASM303615v1 | GCF_003036155.1 | 90550 | 2233663 | 2018/04/05 | 2219 | PRJNA342349 | SAMN06172937 |
| Canada | Bovine milk | strain: SNUC 4042 | ASM303618v1 | GCF_003036185.1 | 59940 | 2324296 | 2018/04/05 | 2349 | PRJNA342349 | SAMN06172936 |
| Canada | Bovine milk | strain: SNUC 3885 | ASM303620v1 | GCF_003036205.1 | 85739 | 2325518 | 2018/04/05 | 2343 | PRJNA342349 | SAMN06172934 |
| Canada | Bovine milk | strain: SNUC 2899 | ASM303621v1 | GCF_003036215.1 | 80140 | 2328216 | 2018/04/05 | 2353 | PRJNA342349 | SAMN06172931 |
| Canada | Bovine milk | strain: SNUC 3611 | ASM303624v1 | GCF_003036245.1 | 108177 | 2269670 | 2018/04/05 | 2278 | PRJNA342349 | SAMN06172933 |
| Canada | Bovine milk | strain: SNUC 3095 | ASM303626v1 | GCF_003036265.1 | 71459 | 2326031 | 2018/04/05 | 2339 | PRJNA342349 | SAMN06172932 |
| Canada | Bovine milk | strain: SNUC 2579 | ASM303628v1 | GCF_003036285.1 | 180607 | 2272423 | 2018/04/05 | 2264 | PRJNA342349 | SAMN06172930 |
| Canada | Bovine milk | strain: SNUC 2460 | ASM303630v1 | GCF_003036305.1 | 93622 | 2291877 | 2018/04/05 | 2290 | PRJNA342349 | SAMN06172927 |
| Canada | Bovine milk | strain: SNUC 2458 | ASM303632v1 | GCF_003036325.1 | 73028 | 2301017 | 2018/04/05 | 2303 | PRJNA342349 | SAMN06172926 |
| Canada | Bovine milk | strain: SNUC 1415 | ASM303634v1 | GCF_003036345.1 | 54166 | 2276884 | 2018/04/05 | 2279 | PRJNA342349 | SAMN06172919 |
| Canada | Bovine milk | strain: SNUC 1344 | ASM303636v1 | GCF_003036365.1 | 1216587 | 2299735 | 2018/04/05 | 2288 | PRJNA342349 | SAMN06172904 |
| Canada | Bovine milk | strain: SNUC 1369 | ASM303637v1 | GCF_003036375.1 | 1216574 | 2294780 | 2018/04/05 | 2288 | PRJNA342349 | SAMN06172910 |
| Canada | Bovine milk | strain: SNUC 1341 | ASM303640v1 | GCF_003036405.1 | 1209492 | 2265099 | 2018/04/05 | 2241 | PRJNA342349 | SAMN06172903 |
| Canada | Bovine milk | strain: SNUC 1405 | ASM303943v1 | GCF_003039435.1 | 19798 | 2305964 | 2018/04/05 | 2353 | PRJNA342349 | SAMN06172918 |
| Canada | Bovine milk | strain: SNUC 1328 | ASM303945v1 | GCF_003039455.1 | 83254 | 2262532 | 2018/04/05 | 2246 | PRJNA342349 | SAMN06172901 |
| Canada | Bovine milk | strain: SNUC 1318 | ASM303949v1 | GCF_003039495.1 | 14484 | 2301241 | 2018/04/05 | 2375 | PRJNA342349 | SAMN06172899 |
| Canada | Bovine milk | strain: SNUC 1139 | ASM303951v1 | GCF_003039515.1 | 84248 | 2307631 | 2018/04/05 | 2318 | PRJNA342349 | SAMN06172896 |
| Canada | Bovine milk | strain: SNUC 1062 | ASM303953v1 | GCF_003039535.1 | 105472 | 2323190 | 2018/04/05 | 2352 | PRJNA342349 | SAMN06172894 |
| Canada | Bovine milk | strain: SNUC 1015 | ASM303955v1 | GCF_003039555.1 | 90250 | 2275620 | 2018/04/05 | 2266 | PRJNA342349 | SAMN06172892 |
| Canada | Bovine milk | strain: SNUC 859 | ASM303957v1 | GCF_003039575.1 | 93198 | 2346321 | 2018/04/05 | 2372 | PRJNA342349 | SAMN06172891 |
| Canada | Bovine milk | strain: SNUC 744 | ASM303959v1 | GCF_003039595.1 | 51687 | 2344809 | 2018/04/05 | 2355 | PRJNA342349 | SAMN06172889 |
| Canada | Bovine milk | strain: SNUC 544 | ASM303961v1 | GCF_003039615.1 | 142289 | 2320835 | 2018/04/05 | 2315 | PRJNA342349 | SAMN06172887 |
| Canada | Bovine milk | strain: SNUC 505 | ASM303963v1 | GCF_003039635.1 | 36160 | 2304652 | 2018/04/05 | 2316 | PRJNA342349 | SAMN06172886 |
| Canada | Bovine milk | strain: SNUC 265 | ASM303965v1 | GCF_003039655.1 | 73446 | 2384669 | 2018/04/05 | 2445 | PRJNA342349 | SAMN06172882 |
| Canada | Bovine milk | strain: SNUC 255 | ASM303967v1 | GCF_003039675.1 | 86381 | 2320517 | 2018/04/05 | 2324 | PRJNA342349 | SAMN06172881 |
| Canada | Bovine milk | strain: SNUC 235 | ASM303969v1 | GCF_003039695.1 | 117483 | 2253881 | 2018/04/05 | 2251 | PRJNA342349 | SAMN06172880 |
| Canada | Bovine milk | strain: SNUC 134 | ASM303971v1 | GCF_003039715.1 | 32785 | 2315875 | 2018/04/05 | 2347 | PRJNA342349 | SAMN06172879 |
| Canada | Bovine milk | strain: SNUC 126 | ASM303973v1 | GCF_003039735.1 | 45460 | 2341454 | 2018/04/05 | 2362 | PRJNA342349 | SAMN06172875 |
| Canada | Bovine milk | strain: SNUC 114 | ASM303975v1 | GCF_003039755.1 | 85713 | 2292203 | 2018/04/05 | 2297 | PRJNA342349 | SAMN06172874 |
| Canada | Bovine milk | strain: SNUC 110 | ASM303977v1 | GCF_003039775.1 | 76246 | 2309112 | 2018/04/05 | 2314 | PRJNA342349 | SAMN06172873 |
| Canada | Bovine milk | strain: SNUC 105 | ASM303979v1 | GCF_003039795.1 | 190681 | 2301023 | 2018/04/05 | 2299 | PRJNA342349 | SAMN06172871 |
| Canada | Bovine milk | strain: SNUC 104 | ASM303981v1 | GCF_003039815.1 | 159851 | 2376116 | 2018/04/05 | 2399 | PRJNA342349 | SAMN06172870 |
| Canada | Bovine milk | strain: SNUC 24 | ASM303983v1 | GCF_003039835.1 | 28627 | 2352853 | 2018/04/05 | 2389 | PRJNA342349 | SAMN06172867 |
| Canada | Bovine milk | strain: SNUC 1015.1 | ASM304001v1 | GCF_003040015.1 | 54560 | 2276835 | 2018/04/05 | 2277 | PRJNA342349 | SAMN06172946 |
| Canada | Bovine milk | strain: SNUC 6071 | ASM304003v1 | GCF_003040035.1 | 77213 | 2306442 | 2018/04/05 | 2311 | PRJNA342349 | SAMN06172945 |
| Canada | Bovine milk | strain: SNUC 4613 | ASM304005v1 | GCF_003040055.1 | 1207057 | 2323362 | 2018/04/05 | 2332 | PRJNA342349 | SAMN06172939 |
| Canada | Bovine milk | strain: SNUC 2487 | ASM304007v1 | GCF_003040075.1 | 67107 | 2268562 | 2018/04/05 | 2258 | PRJNA342349 | SAMN06172929 |
| Canada | Bovine milk | strain: SNUC 2109 | ASM304009v1 | GCF_003040095.1 | 142414 | 2306989 | 2018/04/05 | 2336 | PRJNA342349 | SAMN06172925 |
| Canada | Bovine milk | strain: SNUC 2004 | ASM304011v1 | GCF_003040115.1 | 31049 | 2284377 | 2018/04/05 | 2311 | PRJNA342349 | SAMN06172924 |
| Canada | Bovine milk | strain: SNUC 1445 | ASM304013v1 | GCF_003040135.1 | 50884 | 2372764 | 2018/04/05 | 2421 | PRJNA342349 | SAMN06172920 |
| Canada | Bovine milk | strain: SNUC 1381 | ASM304015v1 | GCF_003040155.1 | 63522 | 2271218 | 2018/04/05 | 2285 | PRJNA342349 | SAMN06172915 |
| Canada | Bovine milk | strain: SNUC 1374 | ASM304017v1 | GCF_003040175.1 | 65464 | 2315145 | 2018/04/05 | 2309 | PRJNA342349 | SAMN06172912 |
| Canada | Bovine milk | strain: SNUC 1363 | ASM304019v1 | GCF_003040195.1 | 75262 | 2456424 | 2018/04/05 | 2515 | PRJNA342349 | SAMN06172909 |
| Canada | Bovine milk | strain: SNUC 1362 | ASM304021v1 | GCF_003040215.1 | 24444 | 2302011 | 2018/04/05 | 2328 | PRJNA342349 | SAMN06172908 |
| Canada | Bovine milk | strain: SNUC 1351 | ASM304023v1 | GCF_003040235.1 | 249827 | 2321532 | 2018/04/05 | 2344 | PRJNA342349 | SAMN06172907 |
| Canada | Bovine milk | strain: SNUC 1350 | ASM304025v1 | GCF_003040255.1 | 1216587 | 2298525 | 2018/04/05 | 2293 | PRJNA342349 | SAMN06172906 |
| Canada | Bovine milk | strain: SNUC 1339 | ASM304027v1 | GCF_003040275.1 | 1164963 | 2311384 | 2018/04/05 | 2296 | PRJNA342349 | SAMN06172902 |
| Canada | Bovine milk | strain: SNUC 1321 | ASM304029v1 | GCF_003040295.1 | 69858 | 2336620 | 2018/04/05 | 2372 | PRJNA342349 | SAMN06172900 |
| Canada | Bovine milk | strain: SNUC 1261 | ASM304031v1 | GCF_003040315.1 | 14148 | 2312972 | 2018/04/05 | 2393 | PRJNA342349 | SAMN06172898 |
| Canada | Bovine milk | strain: SNUC 1063 | ASM304033v1 | GCF_003040335.1 | 108692 | 2339668 | 2018/04/05 | 2335 | PRJNA342349 | SAMN06172895 |
| Canada | Bovine milk | strain: SNUC 803 | ASM304035v1 | GCF_003040355.1 | 51956 | 2286892 | 2018/04/05 | 2287 | PRJNA342349 | SAMN06172890 |
| Canada | Bovine milk | strain: SNUC 277 | ASM304037v1 | GCF_003040375.1 | 24045 | 2283263 | 2018/04/05 | 2307 | PRJNA342349 | SAMN06172883 |
| Canada | Bovine milk | strain: SNUC 133 | ASM304039v1 | GCF_003040395.1 | 38626 | 2364790 | 2018/04/05 | 2419 | PRJNA342349 | SAMN06172878 |
| Canada | Bovine milk | strain: SNUC 132 | ASM304041v1 | GCF_003040415.1 | 26555 | 2333758 | 2018/04/05 | 2383 | PRJNA342349 | SAMN06172877 |
| Canada | Bovine milk | strain: SNUC 129 | ASM304043v1 | GCF_003040435.1 | 266096 | 2343622 | 2018/04/05 | 2353 | PRJNA342349 | SAMN06172876 |
| Canada | Bovine milk | strain: SNUC 107 | ASM304045v1 | GCF_003040455.1 | 105376 | 2301246 | 2018/04/05 | 2298 | PRJNA342349 | SAMN06172872 |
| Canada | Bovine milk | strain: SNUC 91 | ASM304047v1 | GCF_003040475.1 | 74088 | 2321481 | 2018/04/05 | 2345 | PRJNA342349 | SAMN06172869 |
| Canada | Bovine milk | strain: SNUC 65 | ASM304049v1 | GCF_003040495.1 | 32383 | 2334856 | 2018/04/05 | 2372 | PRJNA342349 | SAMN06172868 |
| Canada | Bovine milk | strain: SNUC 4 | ASM304051v1 | GCF_003040515.1 | 269088 | 2341362 | 2018/04/05 | 2330 | PRJNA342349 | SAMN06172865 |
| Canada | Bovine milk | strain: SNUC 6 | ASM304303v1 | GCF_003043035.1 | 181998 | 2302467 | 2018/04/06 | 2295 | PRJNA342349 | SAMN06172866 |
| Canada | Bovine milk | strain: SNUC 2476 | ASM357817v1 | GCF_003578175.1 | 30717 | 2329952 | 2018/09/20 | 2371 | PRJNA342349 | SAMN06172928 |
| Canada | Bovine milk | strain: SNUC 1776 | ASM357818v1 | GCF_003578185.1 | 59060 | 2250281 | 2018/09/20 | 2241 | PRJNA342349 | SAMN06172923 |
| Canada | Bovine milk | strain: SNUC 1603 | ASM357821v1 | GCF_003578215.1 | 84701 | 2364145 | 2018/09/20 | 2434 | PRJNA342349 | SAMN06172922 |
| Canada | Bovine milk | strain: SNUC 1508 | ASM357822v1 | GCF_003578225.1 | 18360 | 2412083 | 2018/09/20 | 2483 | PRJNA342349 | SAMN06172921 |
| Canada | Bovine milk | strain: SNUC 1386 | ASM357826v1 | GCF_003578265.1 | 248425 | 2362855 | 2018/09/20 | 2389 | PRJNA342349 | SAMN06172916 |
| Canada | Bovine milk | strain: SNUC 1379 | ASM357828v1 | GCF_003578285.1 | 24821 | 2266227 | 2018/09/20 | 2292 | PRJNA342349 | SAMN06172914 |
| Canada | Bovine milk | strain: SNUC 1373 | ASM357829v1 | GCF_003578295.1 | 141795 | 2230212 | 2018/09/20 | 2208 | PRJNA342349 | SAMN06172911 |
| Canada | Bovine milk | strain: SNUC 578 | ASM357832v1 | GCF_003578325.1 | 71041 | 2320010 | 2018/09/20 | 2338 | PRJNA342349 | SAMN06172888 |
| Canada | Bovine milk | strain: SNUC 3912 | ASM357843v1 | GCF_003578435.1 | 249419 | 2382376 | 2018/09/20 | 2427 | PRJNA342349 | SAMN06172935 |
| Canada | Bovine milk | strain: SNUC 1387 | ASM357846v1 | GCF_003578465.1 | 1216536 | 2322159 | 2018/09/20 | 2323 | PRJNA342349 | SAMN06172917 |
| Canada | Bovine milk | strain: SNUC 1376 | ASM357848v1 | GCF_003578485.1 | 187495 | 2280142 | 2018/09/20 | 2280 | PRJNA342349 | SAMN06172913 |
| Canada | Bovine milk | strain: SNUC 1348 | ASM357849v1 | GCF_003578495.1 | 249454 | 2368728 | 2018/09/20 | 2391 | PRJNA342349 | SAMN06172905 |
| Canada | Bovine milk | strain: SNUC 1158 | ASM357852v1 | GCF_003578525.1 | 16531 | 2296423 | 2018/09/20 | 2321 | PRJNA342349 | SAMN06172897 |
| Canada | Bovine milk | strain: SNUC 1031 | ASM357853v1 | GCF_003578535.1 | 83760 | 2407142 | 2018/09/20 | 2459 | PRJNA342349 | SAMN06172893 |
| Canada | Bovine milk | strain: SNUC 429 | ASM357854v1 | GCF_003578545.1 | 38634 | 2363128 | 2018/09/20 | 2389 | PRJNA342349 | SAMN06172885 |
| India | Bovine milk | strain: K29 | ASM1914908v1 | GCF_019149085.1 | 237107 | 2329474 | 2021/07/06 | 2300 | PRJNA738617 | SAMN19735783 |
| India | Bovine milk | strain: K17 | ASM1914916v1 | GCF_019149165.1 | 180359 | 2321223 | 2021/07/06 | 2297 | PRJNA738612 | SAMN19735774 |
| India | Bovine milk | strain: K23 | ASM1933423v1 | GCF_019334235.1 | 123121 | 2349003 | 2021/07/24 | 2351 | PRJNA741597 | SAMN19883556 |
| India | Bovine milk | strain: K26 | ASM1942967v1 | GCF_019429675.1 | 107269 | 2340405 | 2021/07/31 | 2363 | PRJNA741602 | SAMN19883479 |
| India | Bovine milk | strain: G29 | ASM2118977v1 | GCF_021189775.1 | 161290 | 2413311 | 2021/12/15 | 2397 | PRJNA636233 | SAMN20568242 |
| India | Bovine milk | strain: KM313 | ASM2119101v1 | GCF_021191015.1 | 1258659 | 2330864 | 2021/12/15 | 2349 | PRJNA636233 | SAMN21849945 |
| India | Bovine milk | strain: KM186 | ASM2119131v1 | GCF_021191315.1 | 248668 | 2337680 | 2021/12/15 | 2357 | PRJNA636233 | SAMN21849938 |
| India | Bovine milk | strain: E47MOW | ASM2119145v1 | GCF_021191455.1 | 249228 | 2352648 | 2021/12/15 | 2381 | PRJNA636233 | SAMN21849937 |
| Norway | Bovine milk | strain: 8383-1 | ASM2136770v1 | GCF_021367705.1 | 409797 | 2316340 | 2022/01/05 | 2337 | PRJNA609060 | SAMN19114534 |
| Norway | Bovine milk | strain: 8317-4 | ASM2136774v1 | GCF_021367745.1 | 249840 | 2297555 | 2022/01/05 | 2292 | PRJNA609060 | SAMN19114533 |
| Russia | Bovine milk | strain: NNSch 2386 | ASM3097154v1 | GCF_030971545.1 | 20568 | 2459241 | 2023/08/29 | 2740 | PRJNA1008453 | SAMN37120351 |
| Somalia | Bovine milk | strain: IVB6200 | ASM2555886v1 | GCF_025558865.1 | 2371745 | 2371745 | 2022/10/04 | 2304 | PRJNA819273 | SAMN26923351 |
| Somalia | Bovine milk | strain: IVB6199 | ASM2555890v1 | GCF_025558905.1 | 2264062 | 2264062 | 2022/10/04 | 2286 | PRJNA819273 | SAMN26923350 |
| Thailand | Bovine milk | strain: M123.2 | ASM3246835v1 | GCF_032468355.1 | 405319 | 2311878 | 2023/10/10 | 2318 | PRJNA987337 | SAMN35977271 |
| Thailand | Bovine milk | strain: M84.1 | ASM3246836v1 | GCF_032468365.1 | 319521 | 2395290 | 2023/10/10 | 2456 | PRJNA987337 | SAMN35977270 |
| Thailand | Bovine milk | strain: M79.3 | ASM3246843v1 | GCF_032468435.1 | 249888 | 2514154 | 2023/10/10 | 2594 | PRJNA987337 | SAMN35977269 |
| Thailand | Bovine milk | strain: M87.4.2 | ASM3246845v1 | GCF_032468455.1 | 168053 | 2371860 | 2023/10/10 | 2396 | PRJNA987337 | SAMN35977267 |
| Thailand | Bovine milk | strain: M24.1 | ASM3246849v1 | GCF_032468495.1 | 249018 | 2373814 | 2023/10/10 | 2411 | PRJNA987337 | SAMN35977266 |
| United State of America | Bovine milk | strain: MU 970 | SCHRMU_9701.0 | GCF_000696815.1 | 258778 | 2344537 | 2014/06/02 | 2336 | PRJNA172066 | SAMN02739851 |
| Brazil | Buffalo | strain: 34B | ASM781495v1 | GCF_007814955.1 | 2369172 | 2369172 | 2019/08/01 | 2332 | PRJNA482667 | SAMN09714551 |
| Brazil | Buffalo milk | strain: 20B | ASM781383v1 | GCF_007813835.1 | 2424566 | 2424566 | 2019/08/01 | 2408 | PRJNA482667 | SAMN09714506 |
| Brazil | Buffalo milk | strain: 17A | ASM781452v1 | GCF_007814525.1 | 2351540 | 2394574 | 2019/08/01 | 2358 | PRJNA482667 | SAMN09714428 |
| China | Chicken | strain: SC25 | ASM1535510v1 | GCF_015355105.1 | 166927 | 2518045 | 2020/11/11 | 2571 | PRJNA648661 | SAMN15643680 |
| China | Chicken | strain: SC23 | ASM1535515v1 | GCF_015355155.1 | 551587 | 2424470 | 2020/11/11 | 2441 | PRJNA648661 | SAMN15643678 |
| China | Chicken | strain: SC24 | ASM1535517v1 | GCF_015355175.1 | 142340 | 2518887 | 2020/11/11 | 2584 | PRJNA648661 | SAMN15643679 |
| China | Chicken | strain: SC20 | ASM1535518v1 | GCF_015355185.1 | 202561 | 2517719 | 2020/11/11 | 2559 | PRJNA648661 | SAMN15643675 |
| China | Chicken | strain: SC21 | ASM1535520v1 | GCF_015355205.1 | 202296 | 2504021 | 2020/11/11 | 2544 | PRJNA648661 | SAMN15643676 |
| China | Chicken | strain: SC22 | ASM1535523v1 | GCF_015355235.1 | 281312 | 2489326 | 2020/11/11 | 2522 | PRJNA648661 | SAMN15643677 |
| China | Chicken | strain: SC18 | ASM1535524v1 | GCF_015355245.1 | 200462 | 2516936 | 2020/11/11 | 2551 | PRJNA648661 | SAMN15643673 |
| China | Chicken | strain: SC19 | ASM1535527v1 | GCF_015355275.1 | 202561 | 2516989 | 2020/11/11 | 2550 | PRJNA648661 | SAMN15643674 |
| China | Chicken | strain: SC17 | ASM1535529v1 | GCF_015355295.1 | 551784 | 2488520 | 2020/11/11 | 2512 | PRJNA648661 | SAMN15643672 |
| China | Chicken | strain: SC15 | ASM1535530v1 | GCF_015355305.1 | 202561 | 2516368 | 2020/11/11 | 2550 | PRJNA648661 | SAMN15643670 |
| China | Chicken | strain: SC16 | ASM1535532v1 | GCF_015355325.1 | 202494 | 2496648 | 2020/11/11 | 2543 | PRJNA648661 | SAMN15643671 |
| China | Chicken | strain: SC14 | ASM1535535v1 | GCF_015355355.1 | 281312 | 2488905 | 2020/11/11 | 2516 | PRJNA648661 | SAMN15643669 |
| China | Chicken | strain: SC10 | ASM1535537v1 | GCF_015355375.1 | 202296 | 2503325 | 2020/11/11 | 2555 | PRJNA648661 | SAMN15643665 |
| China | Chicken | strain: SC13 | ASM1535538v1 | GCF_015355385.1 | 200462 | 2518183 | 2020/11/11 | 2557 | PRJNA648661 | SAMN15643668 |
| China | Chicken | strain: SC11 | ASM1535539v1 | GCF_015355395.1 | 202296 | 2502145 | 2020/11/11 | 2544 | PRJNA648661 | SAMN15643666 |
| China | Chicken | strain: SC12 | ASM1535543v1 | GCF_015355435.1 | 200462 | 2517598 | 2020/11/11 | 2555 | PRJNA648661 | SAMN15643667 |
| China | Chicken | strain: SC9 | ASM1535545v1 | GCF_015355455.1 | 192892 | 2517493 | 2020/11/11 | 2550 | PRJNA648661 | SAMN15643664 |
| China | Chicken | strain: SC8 | ASM1535546v1 | GCF_015355465.1 | 202296 | 2488896 | 2020/11/11 | 2519 | PRJNA648661 | SAMN15643663 |
| China | Chicken | strain: SC7 | ASM1535547v1 | GCF_015355475.1 | 202561 | 2516961 | 2020/11/11 | 2559 | PRJNA648661 | SAMN15643662 |
| China | Chicken | strain: SC6 | ASM1535548v1 | GCF_015355485.1 | 202296 | 2484738 | 2020/11/11 | 2517 | PRJNA648661 | SAMN15643661 |
| China | Chicken | strain: SC5 | ASM1535550v1 | GCF_015355505.1 | 202200 | 2484020 | 2020/11/11 | 2517 | PRJNA648661 | SAMN15643660 |
| China | Chicken | strain: SC4 | ASM1535555v1 | GCF_015355555.1 | 197072 | 2437187 | 2020/11/11 | 2467 | PRJNA648661 | SAMN15643659 |
| China | Chicken | strain: SC3 | ASM1535557v1 | GCF_015355575.1 | 202561 | 2555550 | 2020/11/11 | 2610 | PRJNA648661 | SAMN15643658 |
| China | Chicken | strain: SC2 | ASM1535558v1 | GCF_015355585.1 | 174248 | 2453056 | 2020/11/11 | 2488 | PRJNA648661 | SAMN15643657 |
| China | Chicken | strain: SC1 | ASM1535560v1 | GCF_015355605.1 | 202561 | 2527443 | 2020/11/11 | 2572 | PRJNA648661 | SAMN15643656 |
| United State of America | Chicken | strain: 1401 | ASM1146687v1 | GCF_011466875.1 | 2350748 | 2478658 | 2020/03/19 | 2469 | PRJNA553673 | SAMN13231957 |
| India | Goat | strain: Gar RS 4 | ASM3579207v1 | GCF_035792075.1 | 639072 | 2379964 | 2024/01/17 | 2419 | PRJNA636233 | SAMN29049800 |
| India | Goat | strain: Gar RS 3 | ASM3579211v1 | GCF_035792115.1 | 1215359 | 2340033 | 2024/01/17 | 2328 | PRJNA636233 | SAMN29049798 |
| Brazil | Goat milk | strain: 76ME | ASM2665024v1 | GCF_026650245.1 | 2256804 | 2256804 | 2022/12/05 | 2225 | PRJNA769457 | SAMN22137267 |
| China | Human | strain: PS2Phumoskin1 | ASM4752843v1 | GCF_047528435.1 | 1197800 | 2307260 | 2025/02/09 | 2321 | PRJNA1021388 | SAMN46432922 |
| Canada | Laboratory isolate ATCC | strain: ATCC 43764 | ASM1777514v1 | GCF_017775145.1 | 250031 | 2274307 | 2021/04/05 | 2252 | PRJNA630769 | SAMN14848453 |
| South Africa | Pig | strain: ST161 | ASM3184661v1 | GCF_031846615.1 | 1339295 | 2434252 | 2023/09/23 | 2413 | PRJNA1018240 | SAMN37429359 |
| South Africa | Pig | strain: ST159 | ASM3184666v1 | GCF_031846665.1 | 533687 | 2337691 | 2023/09/23 | 2288 | PRJNA1018240 | SAMN37429355 |
| South Africa | Pig | strain: ST157 | ASM3184667v1 | GCF_031846675.1 | 331844 | 2296366 | 2023/09/23 | 2275 | PRJNA1018240 | SAMN37429357 |
| South Africa | Pig | strain: ST151 | ASM3184670v1 | GCF_031846705.1 | 533686 | 2343140 | 2023/09/23 | 2292 | PRJNA1018240 | SAMN37429354 |
| South Africa | Pig | strain: ST150 | ASM3184672v1 | GCF_031846725.1 | 297155 | 2272030 | 2023/09/23 | 2240 | PRJNA1018240 | SAMN37429358 |
| South Africa | Pig | strain: ST160 | ASM3184675v1 | GCF_031846755.1 | 520712 | 2307655 | 2023/09/23 | 2271 | PRJNA1018240 | SAMN37429353 |
| South Africa | Pig | strain: ST158 | ASM3184678v1 | GCF_031846785.1 | 378096 | 2352050 | 2023/09/23 | 2314 | PRJNA1018240 | SAMN37429356 |
| South Africa | Pig | strain: ST155 | ASM3184693v1 | GCF_031846935.1 | 307562 | 2342193 | 2023/09/23 | 2308 | PRJNA1018240 | SAMN37429343 |
| South Africa | Pig | strain: ST153 | ASM3184697v1 | GCF_031846975.1 | 248943 | 2318088 | 2023/09/23 | 2278 | PRJNA1018240 | SAMN37429342 |
| South Africa | Pig | strain: ST154 | ASM3184699v1 | GCF_031846995.1 | 276534 | 2364429 | 2023/09/23 | 2331 | PRJNA1018240 | SAMN37429341 |
| South Africa | Pig | strain: ST156 | ASM3184701v1 | GCF_031847015.1 | 538126 | 2337974 | 2023/09/23 | 2290 | PRJNA1018240 | SAMN37429340 |
| South Africa | Pig | strain: ST152 | ASM3184703v1 | GCF_031847035.1 | 249370 | 2265120 | 2023/09/23 | 2226 | PRJNA1018240 | SAMN37429339 |
| United State of America | Pig | strain: NCTC 10530 | ASM290194v1 | GCF_002901945.1 | 55359 | 2276768 | 2018/01/27 | 2286 | PRJNA339206 | SAMN05978023 |
| United Kingdom | Pig | strain: NCTC10530 | 41315_F01 | GCF_900458195.1 | 2280656 | 2310776 | 2018/08/01 | 2270 | PRJEB6403 | SAMEA3518015 |
| Unknown | Pig | strain: DSM 20454 | ASM2902462v1 | GCF_029024625.1 | 2280008 | 2300787 | 2023/03/08 | 2258 | PRJNA936091 | SAMN33336518 |
| United State of America | Soil | strain: SDA1 | ASM700420v1 | GCF_007004205.1 | 1201589 | 2279093 | 2019/07/15 | 2272 | PRJNA552487 | SAMN12211899 |
| Unknown | Unknown | strain: CCM 3387 | ASM1463574v1 | GCF_014635745.1 | 1217900 | 2278936 | 2020/09/11 | 2261 | PRJDB10511 | SAMD00244877 |


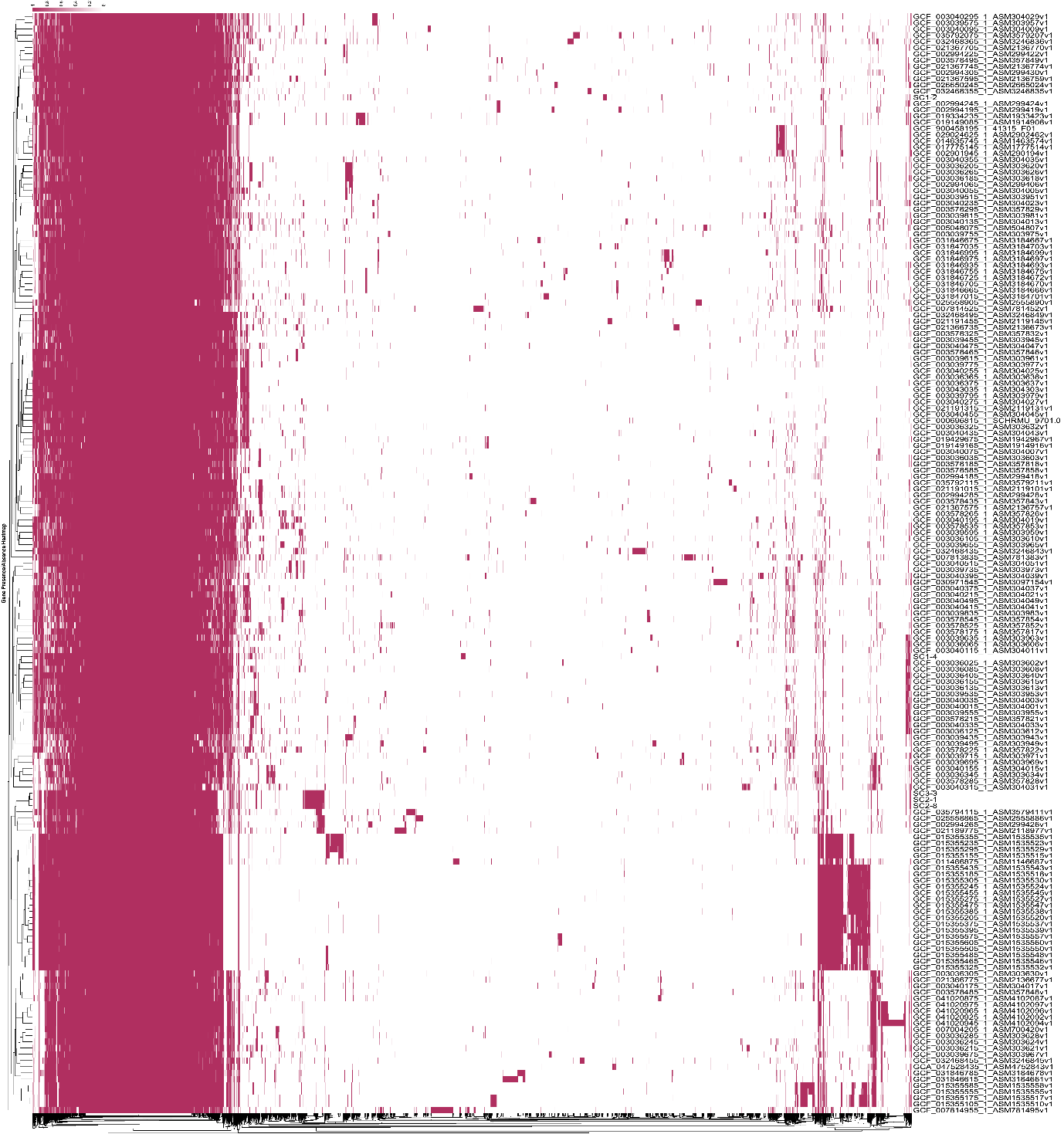


**Supplementary Figure S1:** The pan-genome of the global 177 *Staphylococcus chromogenes*, including the five sequenced genomes in this study from bovine mastitis. The phylogenetic tree heatmap is based on gene present/absent.
