# Supplementary material for: Population structure, resistome, and virulome of Staphylococcus chromogenes strains from milk of subclinical bovine mastitis in South Africa
Source: Front Cell Infect Microbiol. 2025 Aug 22;15:1654546. doi: 10.3389/fcimb.2025.1654546 (PMC12411441; doi:10.3389/fcimb.2025.1654546)
Supplement: Supplementary file 2 [file DataSheet2.docx]

**Population structure, resistome, and virulome of *Staphylococcus chromogenes* strains from milk of subclinical bovine mastitis in South Africa**

**Khasapane N.G^1^, Nkhebenyane S.J^1^, Thekisoe O^2^, Ramatla T.^1^ Lekota K.E^2^**

^1^ Centre for Applied Food Safety and Biotechnology, Department of Life Sciences, Central University of Technology, 1 Park Road, Bloemfontein, 9300, South Africa

^2^ Unit for Environmental Sciences and Management, North-West University, Potchefstroom, 2531, South Africa

***Correspondence:**Khasapane
[nkhasapane@cut.ac.za](mailto:nkhasapane@cut.ac.za)

**Commands used for bioinformatics analysis**

**FastQC** for quality control to check raw sequence data. These command was used:

fastqc sample_R1.fastq -o fastqc_output/

**Trimmomatic v0.39** command used:

trimmomatic PE -phred33 -threads 4 \

sample_R1.fastq.gz sample_R2.fastq.gz \

sample_R1_paired.fastq.gz sample_R1_unpaired.fastq.gz \

sample_R2_paired.fastq.gz sample_R2_unpaired.fastq.gz \

ILLUMINACLIP:TruSeq3-PE.fa:2:30:10 \

LEADING:3 TRAILING:3 SLIDINGWINDOW:4:15 MINLEN:36

SKESA is a de novo assembler for bacterial genomes based on Illumina reads.

skesa --fastq input_reads.fastq --contigs_out skesa_output.fasta --threads 8

**Shovill v1.1.0**

Shovill is a fast pipeline for assembling bacterial genomes from Illumina paired-end reads, wrapping SPAdes.

shovill --R1 reads_1.fastq --R2 reads_2.fastq --outdir shovill_out --depth 100 --cpus 8

**3. QUAST v5.0.2**

QUAST evaluates genome assemblies by generating summary statistics.

quast.py assembly.fasta -o quast_output --threads 8

**CheckM**

CheckM assesses genome completeness and contamination based on lineage-specific marker genes

checkm lineage_wf -x fasta input_folder checkm_output -t 8

**GTDB-Tk v1.7.0**

GTDB-Tk assigns standardized taxonomy based on the Genome Taxonomy Database.

gtdbtk classify_wf --genome_dir input_genomes/ --out_dir gtdbtk_output --cpus 8

**Anvio v8.0.1**

Generate Contigs Databases (one per genome):

anvi-gen-contigs-database -f genome1.fasta -o genome1.db -n 'Genome 1'

Generate a Genomes Storage Database

anvi-gen-genomes-storage -i genomes-info.txt -o genomes-storage.db

name contigs_db_path

Sc1 /path/to/genome1.db

Sc2 /path/to/genome2.db

For ANI analysis: anvi-compute-genome-similarity -e genomes-info.txt -o ani_output --program pyANI --pan-mode --matrix-format both --num-threads 8

Visualize the ANI Matrix:

anvi-display-pan -g genomes-storage.db -p pan-db/PROFILE.db

**ABRicate Pipeline** (with --mincov 70 and --minid 70)

abricate --mincov 70 --minid 70 --db ncbi input_genome.fasta > abricate_output.tab

To run on multiple files in a directory

for file in *.fasta; do

abricate --mincov 70 --minid 70 --db vfdb $file > ${file%.fasta}_abricate.txt

done

The –db were substituted with different databases that included i.e. plasmidfinder, and CARD

**AMRFinderPlus** (with --min_cov and --identity_threshold)

amrfinder -n input_genome.fasta --organism bacteria --min_cov 0.7 --identity_threshold 0.7 -o amrfinder_output.tsv

For **Prokka** v1.14 to annotate the genomes:

prokka input_genome.fasta --outdir prokka_output --prefix sample_name --cpus 8

For **roary** to compute pangenome, this command was used

roary -e -n -v -p 8 *.gff

**Gubbins v3.4** to identify and filter recombination in bacterial genome alignments:

run_gubbins.py --prefix gubbins_output --threads 8 alignment.aln

**IQ-TREE v1.6.10** to generate phylogenetic trees, we used gamma distribution using the following commands:

iqtree -s alignment.fasta -m GTR+G -bb 1000 -nt 8

Pangenome visualization on R script:

library(c("tidyverse", "pheatmap"))  # Run only once

library(tidyverse)

library(pheatmap)

**Read and Transform the Data**

# Load the Roary presence/absence file

roary_data <- read.csv("gene_presence_absence.csv", check.names = FALSE)

# Extract the gene name and strain columns (typically columns from 15 onwards are strain-specific)

strain_data <- roary_data[, c(1, 15:ncol(roary_data))]  # 1 = Gene, others = isolate columns

# Convert the data to long format for easier manipulation

long_data <- strain_data %>%

  pivot_longer(-Gene, names_to = "Strain", values_to = "Presence") %>%

  mutate(Presence = ifelse(Presence == "", 0, 1))  # Empty cell = 0, others = 1

# Convert back to wide format

binary_matrix <- long_data %>%

  pivot_wider(names_from = Strain, values_from = Presence) %>%

  column_to_rownames("Gene")

# Basic heatmap

pheatmap(binary_matrix,

         cluster_rows = TRUE,

         cluster_cols = TRUE,

         show_rownames = FALSE,

         fontsize_col = 10,

         color = colorRampPalette(c("white", " darkgreen "))(100),

         main = "Gene Presence/Absence Heatmap")
